# Transcription factor profiling identifies Sox9 as regulator of proliferation and differentiation in corneal epithelial stem/progenitor cells

**DOI:** 10.1038/s41598-018-28596-3

**Published:** 2018-07-06

**Authors:** Johannes Menzel-Severing, Matthias Zenkel, Naresh Polisetti, Elisabeth Sock, Michael Wegner, Friedrich E. Kruse, Ursula Schlötzer-Schrehardt

**Affiliations:** 1 Department of Ophthalmology, Friedrich-Alexander-Universität Erlangen-Nürnberg, Erlangen, Germany; 2 Institut für Biochemie, Emil-Fischer-Zentrum, Friedrich-Alexander-Universität Erlangen-Nürnberg, Erlangen, Germany

## Abstract

Understanding transcription factor (TF) regulation of limbal epithelial stem/progenitor cells (LEPCs) may aid in using non-ocular cells to regenerate the corneal surface. This study aimed to identify and characterize TF genes expressed specifically in LEPCs isolated from human donor eyes by laser capture microdissection. Using a profiling approach, preferential limbal expression was found for SoxE and SoxF genes, particularly for Sox9, which showed predominantly cytoplasmic localization in basal LEPCs and nuclear localization in suprabasal and corneal epithelial cells, indicating nucleocytoplasmic translocation and activation during LEPC proliferation and differentiation. Increased nuclear localization of Sox9 was also observed in activated LEPCs following clonal expansion and corneal epithelial wound healing. Knockdown of *SOX9* expression in cultured LEPCs by RNAi led to reduced expression of progenitor cell markers, e.g. keratin 15, and increased expression of differentiation markers, e.g. keratin 3. Furthermore, *SOX9* silencing significantly suppressed the proliferative capacity of LEPCs and reduced levels of glycogen synthase kinase 3 beta (GSK-3ß), a negative regulator of Wnt/ß-catenin signaling. Sox9 expression, in turn, was significantly suppressed by treatment of LEPCs with exogenous GSK-3ß inhibitors and enhanced by small molecule inhibitors of Wnt signaling. Our results suggest that Sox9 and Wnt/ß-catenin signaling cooperate in mutually repressive interactions to achieve a balance between quiescence, proliferation and differentiation of LEPCs in the limbal niche. Future molecular dissection of Sox9-Wnt interaction and mechanisms of nucleocytoplasmic shuttling of Sox9 may aid in improving the regenerative potential of LEPCs and the reprogramming of non-ocular cells for corneal surface regeneration.

## Introduction

The cornea forms the most anterior anatomical structure of the eye and has been described as our “window to the world”. Its functions rely heavily on the presence of an intact corneal epithelium^1^. The currently prevailing notion is that unipotent, adult epithelial stem and progenitor cells are responsible for corneal epithelial homeostasis and repair. These are located within a stem cell niche at the transition zone between cornea and sclera, the limbus^2^. A number of different disease entities are held responsible for a deficiency in limbal epithelial stem/progenitor cells (LEPCs), which may lead to painful loss of vision^3^. To provide efficient treatment in cases of unilateral limbal stem cell deficiency, autologous limbal epithelial cells (including stem/progenitor cells) from the healthy contralateral eye can be expanded through *ex vivo* culture and transplanted to the diseased eye^4^. However, the availability of autologous limbal epithelial cells for transplantation is limited, particularly in patients with systemic and/or bilateral corneal disease. To avoid the need for allogeneic transplantation, research efforts have been directed towards the use of progenitor cells from non-ocular sources^5^. Direct transdifferentiation of these cells into a corneal epithelial phenotype or the use of induced pluripotent stem cells (iPSC) have been proposed^6,7^.

Transcription factors (TFs) are key players both in establishing pluripotency and in directing cells towards a new lineage^8^. It is also well established that TFs can play important roles both in pathogenesis and therapy of limbal stem cell deficiency. One example is aniridia-related keratopathy, which is a genetic disorder that stems from haploinsufficiency of the *PAX6* gene^9^. This gene encodes a transcription factor that is crucial for eye development^10^. Also, Rama and co-workers have shown that cultured limbal epithelial grafts will be clinically more successful, if they contain more than 3% of cells that stain brightly for the transcription factor p63^11^. Hence, efforts to dissect TF networks in corneal epithelial cells and in cells of the limbal stem cell compartment may aid in improving the efficacy of emerging therapeutic approaches^6,7^.

It has been suggested that gene expression profiling and comparison of different ocular surface epithelial areas may aid to identify relevant subsets of genes and expression patterns^12^. We have therefore performed a comprehensive screening to identify differentially expressed TFs in human basal limbal stem/progenitor and basal corneal epithelial cells. Our data suggest elevated expression of members of the “*Sry*-related high-mobility group box” (Sox) gene family in LEPCs. Sox genes encode TFs that regulate cell fate and differentiation during development and adult tissue homeostasis^13,14^. Here, we identify *SOX9* to represent the predominant TF expressed in LEPCs. Sox9 localizes to the cytoplasm of basal stem/progenitor cells at the limbus and to cell nuclei of suprabasal and corneal epithelial cells, indicating nucleocytoplasmic shuttling and activation during LEPC proliferation and differentiation. Sox9 upregulation and increased nuclear localization is also observed during LEPC clonal expansion and corneal epithelial wound healing *in vitro*. By employing RNA interference, we further show that Sox9 is essential for promoting LEPC proliferation and lineage commitment without inducing terminal differentiation. Finally, we provide evidence that Sox9 and canonical Wnt/ß-catenin signaling can interact in mutually repressive associations to achieve a balance between quiescence, proliferation and differentiation of LEPCs in the limbal niche.

## Results

### Transcription factor gene expression profiling

First, we assessed differential TF gene expression in LEPC clusters versus basal (central) corneal epithelial cell populations (BCECs) obtained by Laser Capture Microdissection (LCM; n = 5). Quality control of amplified RNA and purity of dissected cell populations were assessed as described previously^15^. Pre-manufactured RT^2^ profiler PCR arrays were used to determine expression levels of 84 TF genes (for full listing, see Supplementary Table 1) in pairs of samples. Table 1 lists all 29 genes for which expression was detected at a reliable level (i.e., by a cycle threshold of ≤35 in both limbal and central corneal samples) and/or differential expression was observed. Genes were considered as differentially expressed when their expression levels exceeded a two-fold difference in all five specimens analysed. This was the case in four genes, which were significantly upregulated in LEPC clusters compared to BCECs (*DACH1, HOXA11, PPARG, SOX9*) and 11 genes that were downregulated (*FOXP2, RB1, MSX2, JUN, PCNA, SP1, SIX2, PAX6, FOXP3, SMAD2, FOXP1*). All genes for which array screening indicated upregulation in LEPC clusters were validated using specific qRT-PCR assays. Due to limited sample material, only 5 out of 11 down-regulated genes were exemplarily validated. Results are also shown in Table 1. Validation confirmed that *SOX9* was the highest upregulated gene in LEPC clusters compared to BCECs with a fold change of 112.7, followed by *PPARG* (29.3), *DACH1* (8.5) and *HOXA11* (7.2).

**Table 1:** Differentially expressed genes in limbal epithelial stem/progenitor cell clusters compared to basal corneal epithelial cells isolated by laser capture microdissection (n = 5).

Gene name	Gene symbol	Fold change (mean ± standard deviation)	
		RT^2^ Profiler PCR array	qRT-PCR primer assays
Dachshund family transcription factor 1	*DACH1*	73.2 ± 27.3*	8.5 ± 1.3*
Homeobox A11	*HOXA11*	66.3 ± 24.5*	7.2 ± 1.8*
Peroxisome proliferator-activated receptor gamma	*PPARG*	35.36 ± 15.6*	29.3 ± 4.6*
Sex determining region Y-box 9	*SOX9*	29.5 ± 13.4*	112.7 ± 21.1**
Forkhead box P2	*FOXP2*	−33.6 ± 5.7*	−2.6 ± 0.7
Retinoblastoma susceptibility protein	*RB1*	−13.1 ± 6.1*	NT
Msh homeobox 2	*MSX2*	−11.1 ± 5.5*	NT
Jun proto-oncogene	*JUN*	−8.5 ± 7.6	NT
Proliferating cell nuclear antigen	*PCNA*	−7.8 ± 3.5*	−2.1 ± 0.2*
Sp1 transcription factor	*SP1*	−6.8 ± 4.1	NT
SIX homeobox 2	*SIX2*	−5.8 ± 5.5	NT
Paired box 6	*PAX6*	−5.4 ± 3.3	ND
Forkhead box P3	*FOXP3*	−4.5 ± 2.7*	−6.5 ± 1.6*
SMAD family member 2	*SMAD2*	−3.6 ± 1.4*	NT
Forkhead box P1	*FOXP1*	−2.6 ± 0.6*	NT
Enhancer of zeste homolog 2	*EZH2*	ND	NT
Kruppel-like Factor 2	*KLF2*	ND	NT
Kruppel-like Factor 4	*KLF4*	ND	NT
V-myc avian myelocytomatosis viral oncogene homolog	*MYC*	ND	NT
Nuclear factor of activated T-cells 1	*NFATC1*	ND	NT
Notch2	*NOTCH2*	ND	NT
Nuclear receptor subfamily 2 group F member 2	*NR2F2*	ND	NT
POU domain, class 5 homeobox 1	*POU5F1*	ND	NT
Runt related transcription factor 1	*RUNX1*	ND	NT
Sex determining region Y-box 6	*SOX6*	ND	ND
Signal transducer and activator of transcription 1	*STAT1*	ND	NT
Signal transducer and activator of transcription 3	*STAT3*	ND	NT
Werner syndrome RecQ like helicase	*WRN*	ND	NT
GATA binding protein 6	*GATA6*	ND	NT

Asterisks indicate statistical significance (*p < 0.05; p < 0.005). Abbreviations: ND, no difference; NT, not tested.

### Sox family gene expression profiling

Because TF profiling suggested pronounced differential expression of Sox family member *SOX9*, further analysis concentrated on the Sox family of TFs. We used specific qRT-PCR assays to analyse expression of all 20 Sox genes in basal limbal and corneal epithelial cells isolated by LCM (n = 5). Table 2 summarises these data. The prototype Sox gene, *SRY*, showed no differential expression between LEPCs and BCECs. Genes of the SoxB1 (*SOX1, SOX2, SOX3*) and SoxB2 (*SOX14*, *SOX21*) groups were not detected. Of the SoxC group (*SOX4*, *SOX11*, *SOX12*), only *SOX4* was detected at a slightly higher level in LEPCs than in BCECs. In the SoxD group, *SOX5* and *SOX13* were differentially expressed between LEPC and BCEC, while *SOX6* showed no differential expression between both cell populations. In the SoxE (*SOX8*, *SOX9*, *SOX10*) and SoxF (*SOX7*, *SOX17*, *SOX18*) groups, all genes were differentially expressed between LEPCs and BCECs, i.e., expressed more strongly in LEPCs than in BCECs. Here, the strongest differences were observed for *SOX9, SOX10* and *SOX8*, which showed significantly higher expression levels (90- to 112-fold) in LEPCs than in BCECs. The SoxG gene *SOX15* was not detected, and expression of the SoxH gene *SOX30* was lower in LEPCs than in BCECs.

**Table 2:** Differential expression of SOX family genes in limbal epithelial progenitor cell clusters compared to basal corneal epithelial cells isolated by laser capture microdissection (n = 5).

Sox group	Gene name	Gene symbol	Fold change (mean ± standard deviation)
SoxA	Sex determining region Y	*SRY*	ND
SoxB1	Sex determining region 1	*SOX1*	UD
	Sex determining region 2	*SOX2*	UD
	Sex determining region 3	*SOX3*	UD
SoxB2	Sex determining region 14	*SOX14*	UD
	Sex determining region 21	*SOX21*	UD
SoxC	Sex determining region 4	*SOX4*	2.2 ± 0.1*
	Sex determining region 11	*SOX11*	UD
	Sex determining region 12	*SOX12*	UD
SoxD	Sex determining region 5	*SOX5*	13.5 ± 2.3**
	Sex determining region 6	*SOX6*	ND
	Sex determining region 13	*SOX13*	−2.9 ± 0.3*
SoxE	Sex determining region 8	*SOX8*	90.9 ± 18.6**
	Sex determining region 9	*SOX9*	112.7 ± 21.1**
	Sex determining region 10	*SOX10*	96.4 ± 14.2**
SoxF	Sex determining region 7	*SOX7*	8.8 ± 1.0*
	Sex determining region 17	*SOX17*	58.5 ± 16.2*
	Sex determining region 18	*SOX18*	13.9 ± 2.6**
SoxG	Sex determining region 15	*SOX15*	ND
SoxH	Sex determining region 30	*SOX30*	−19.6 ± 5.2*

Asterisks indicate statistical significance (*p < 0.05; **p < 0.005). Abbreviations: UD, undetected; ND, no difference.

**Figure 1:**
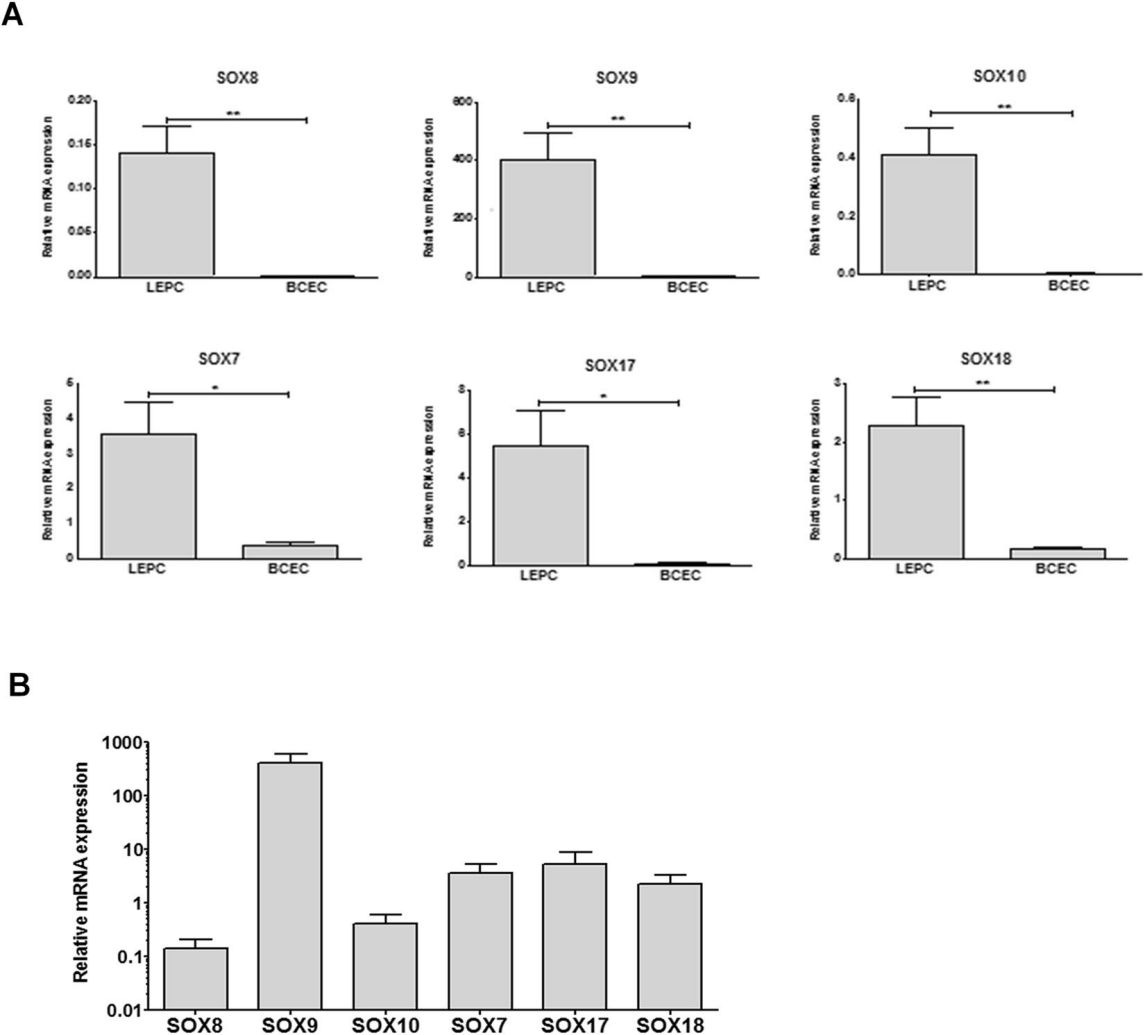
Expression analysis of SoxE and SoxF family members in limbal epithelial progenitor cell (LEPC) clusters compared with basal corneal epithelial cells (BCEC) isolated by laser capture microdissection. Relative expression levels were determined by quantitative real-time polymerase chain reaction (qRT-PCR) primer assays and normalized against GAPDH. Data are expressed as means (2^−∆CT^ × 1,000) ± SD (n = 5); *p < 0.05, **p < 0.005, unpaired *t*-test. (**A**) Relative expression in LEPC compared with BCEC. (**B**) Relative expression (logarithmic scale) of Sox isoforms in LEPC clusters.

Although all SoxE and SoxF family members showed significantly higher expression levels in LEPCs than in BCECs (Fig. 1A), *SOX9* represented the most prominent gene among the identified set of differentially expressed Sox genes in LEPC (Fig. 1B).

### Localization of SoxE proteins *in situ*

**Figure 2:**
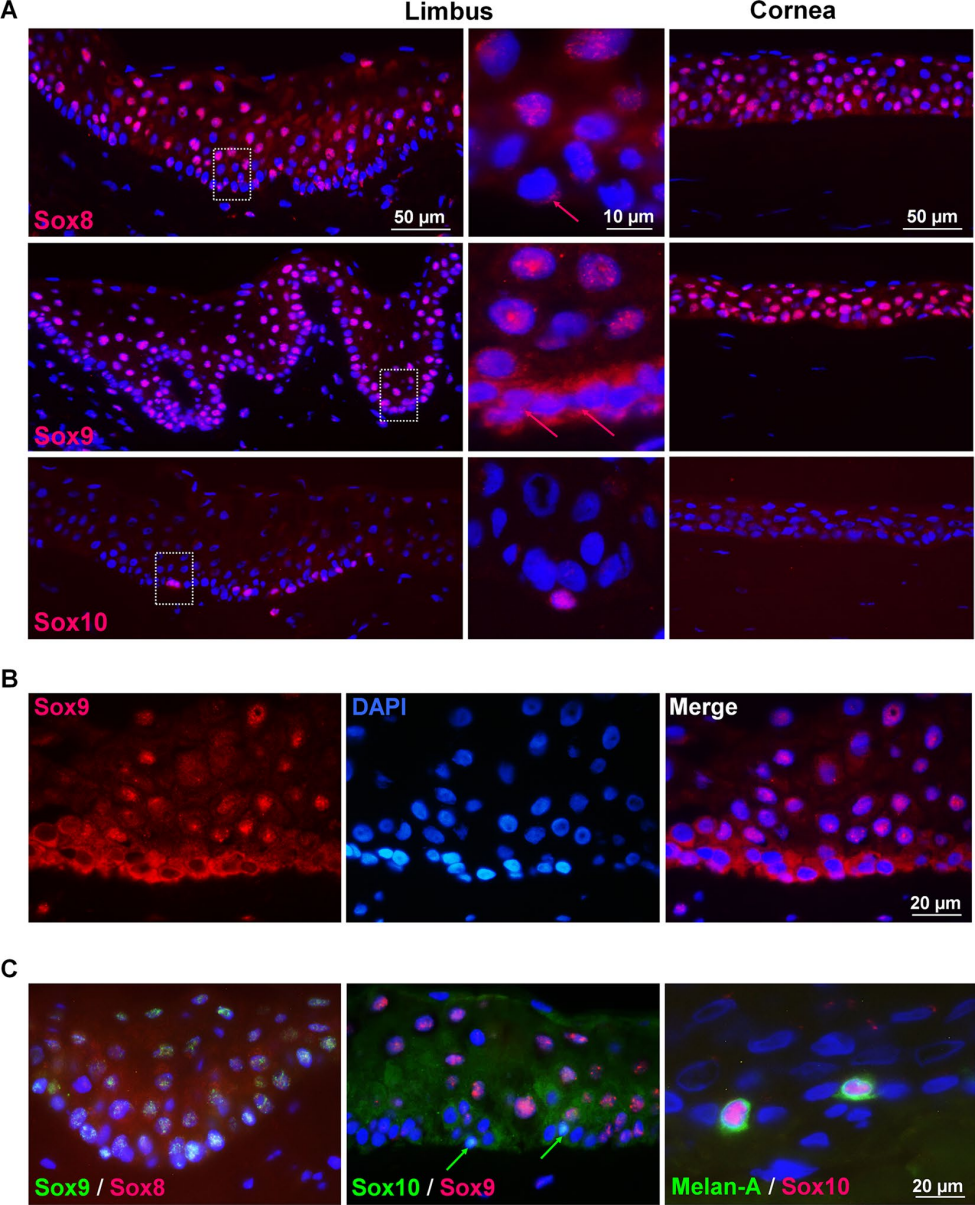
Immunohistochemical localization of SoxE family members in corneoscleral tissue sections. (**A**) Immunofluorescence microscopy demonstrates nuclear staining for Sox8 (clone 4E4.1) and Sox9 (clone 3C10) in suprabasal epithelial cells at the limbus (left column) and central cornea (right column), whereas Sox10 (clone BC34) is confined to few cells in the basal limbal epithelium. Higher magnification images of basal limbal regions (middle column), as indicated by boxed areas, show differential cytoplasmic (arrows) and nuclear localization of Sox8 and Sox9 in basal and suprabasal limbal epithelial cells. (**B**) High magnification images of individual channels show cytoplasmic localization of Sox9 in basal stem/progenitor cell clusters and nuclear localization in suprabasal limbal epithelial cells. (**C**) Double labeling experiments show nuclear co-localization of Sox8 (rabbit IgG) and Sox9 (clone 3C10) (left), distinct localization of Sox9 (clone 3C10) and Sox10 (rabbit IgG) (middle), and localization of Sox10 (clone BC34) to Melan A-positive cells (right). Nuclear counterstaining: DAPI. Individual channels of double labeling experiments are shown in Supplementary Fig. 1.

Based on the gene expression data, members of the SoxE and SoxF groups were selected for further analysis by immunolabeling of corneoscleral tissue sections to confirm their differential expression patterns on protein level (n = 10). Immunostaining for Sox7, Sox17, Sox18 (SoxF group) showed neither pronounced nor preferential localization in LEPC populations at the limbus (data not shown). In contrast, a marked nuclear localization pattern could be observed in limbal and corneal epithelia after staining for Sox8, Sox9 and Sox10 (SoxE group) (Fig. 2A). Immunolocalization of Sox8 was largely confined to nuclei of suprabasal limbal and corneal epithelial cells, whereas it was only weakly expressed in the cytoplasm of basal LEPCs. In addition to a similar nuclear staining pattern, Sox9 was also markedly expressed in the cytoplasm of basal LEPC clusters at the limbus. In contrast, Sox10 was observed only in a small number of cell nuclei in the basal limbal epithelium and occasionally in the subepithelial limbal stroma, but not in the central cornea. The differing subcellular localization between basal stem/progenitor cells (i.e., cytoplasmic) and suprabasal differentiating cells (i.e., nuclear) was most pronounced for Sox9 (Fig. 2B), indicating nucleo-cytoplasmic shuttling of Sox9 during proliferation and early differentiation of LEPC. Co-labeling experiments of the limbal distribution of SoxE proteins showed that Sox8 co-localized with Sox9 in cell nuclei of basal and suprabasal epithelial cells, whereas expression of Sox8/Sox9 and that of Sox10 did not overlap (Fig. 2C, Supplementary Fig. 1). Instead, Sox10-positive cells also expressed Melan-A characterizing them as melanocytes in the basal limbal epithelium.

**Figure 3:**
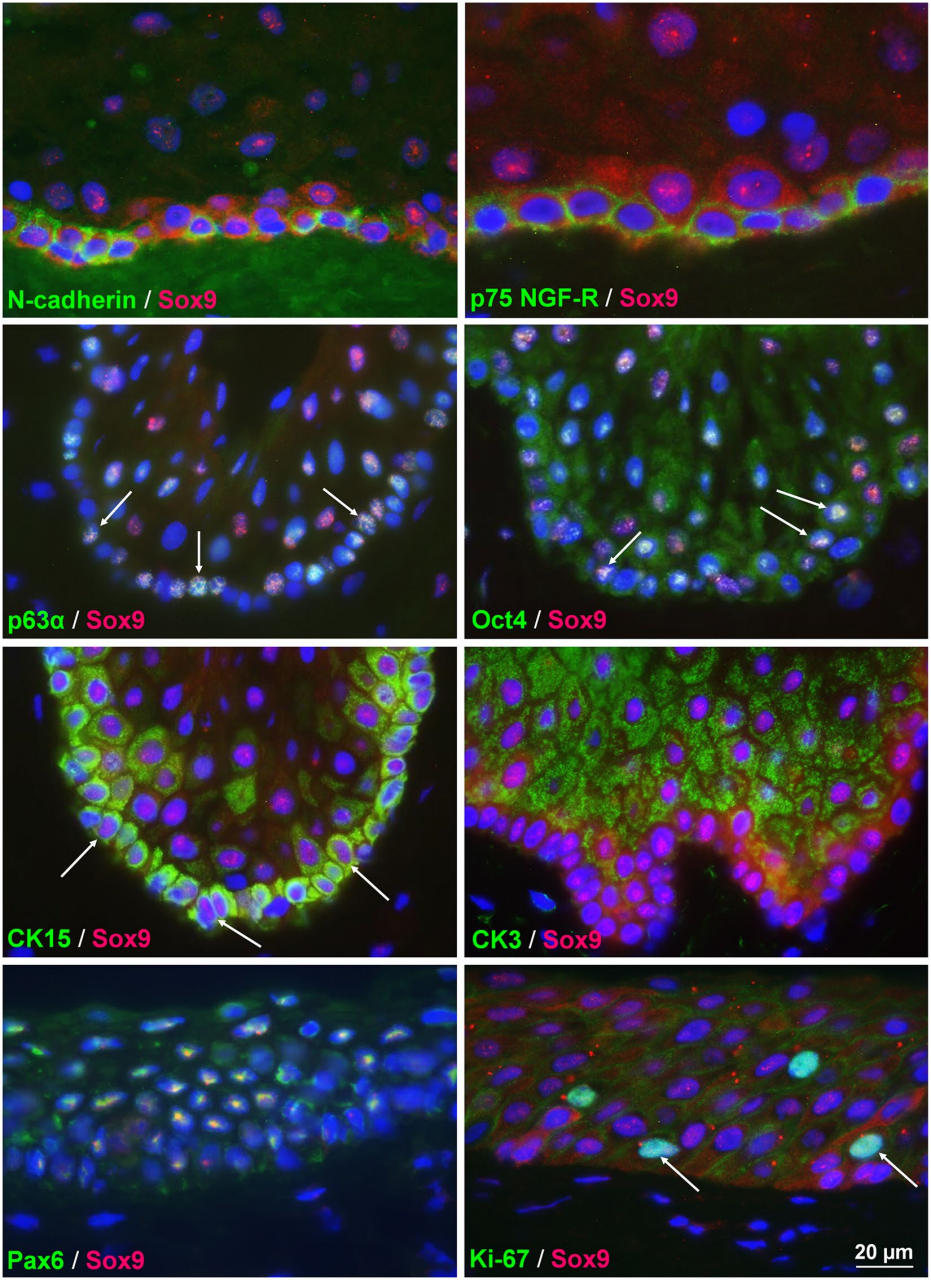
Co-localisation of Sox9 with markers related to progenitor cell phenotype, differentiation and proliferation in the limbal epithelium. Double-labelling demonstrates co-localisation (arrows) of cytoplasmic Sox9 (red) with the stem/progenitor cell markers (green) N-cadherin, p75 nerve growth factor receptor (NGF-R), p63α, Oct4 and cytokeratin (CK) 15) in basal epithelial cells at the limbus. Suprabasal epithelial cells revealed co-localisation of nuclear Sox9 (red) with differentiation-related markers (green) CK3 and Pax6 as well as proliferation-related marker Ki-67. Sox9 monoclonal mouse antibody (clone 3C10) was used for double labelling experiments with polyclonal antibodies against Oct4, p63α, Pax6 and Ki-67, Sox9 polyclonal rabbit antibody (1) was used for double labelling experiments with monoclonal antibodies against p75 NGF-R, N-cadherin, CK3 and CK15. Nuclear counterstaining: DAPI. Individual channels of all double labelling experiments are shown in Supplementary Fig. 2.

Given the low expression levels and the assumed redundancy of Sox8 with Sox9 as well as the obvious restriction of Sox10 expression to melanocytes, Sox9 was selected for more detailed analyses in the limbal stem cell compartment. In double labeling experiments using known limbal epithelial progenitor and corneal epithelial differentiation markers, co-localization was observed between cytoplasmic Sox9 and putative LEPC markers, such as N-cadherin, p75 nerve growth factor receptor, p63α, Oct-4, and keratin 15, in basal limbal epithelial cells (Fig. 3, Supplementary Fig. 2). Co-localization of nuclear Sox9 with differentiation markers, such as keratin 3 and Pax6, was only seen in suprabasal limbal epithelial cells. Co-localization was also occasionally observed between nuclear Sox9 and the proliferation-associated marker Ki-67 in suprabasal cells.

### Sox9 expression during limbal epithelial cell expansion and wound healing *in vitro*

**Figure 4:**
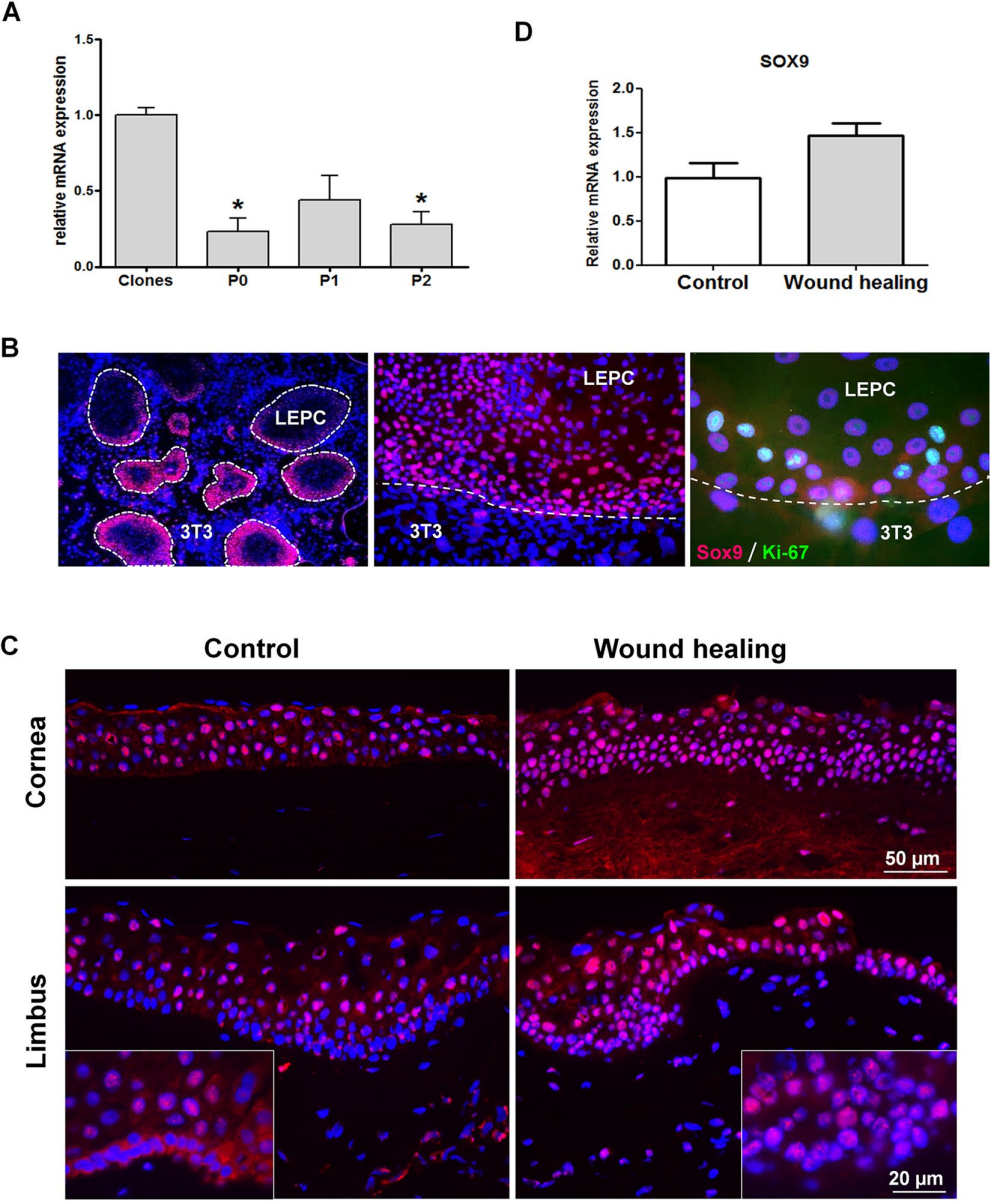
Expression of Sox9 during limbal epithelial cell expansion and wound healing *in vitro*. (**A**) Relative expression levels of Sox9 in cultured limbal epithelial cells expanded as clones on a 3T3 feeder layer or as feeder-free monolayer in passage (P) 0 to P2. Expression levels were determined by quantitative real-time polymerase chain reaction (qRT-PCR) primer assays and normalized against GAPDH. Data are expressed as means (2^−∆CT^ × 1,000) ± SD (n = 3) relative to clonal cultures; *p < 0.01, unpaired *t*-test. (**B**) Limbal epithelial cell (LEPC) clones (dashed lines) on 3T3 feeder cells (3T3) stain positively for Sox9 (clone 3C10, red), preferentially towards the proliferating border of the clones; Sox9-expressing cells partly co-localize with Ki-67 (green). Nuclear staining: DAPI. (**C**) Immunofluorescent staining shows increased levels of nuclear Sox9 (clone 3C10) in basal/suprabasal limbal epithelial cells and central corneal epithelial cells after epithelial debridement and regeneration compared to unwounded control corneas (the background fluorescence seen in the central corneal stroma may be attributed to the epithelial debridement allowing media and serum components to infiltrate the stroma during the wound healing process). Higher magnification images of limbal epithelial progenitor cell clusters show increased nuclear localization of Sox9 in basal epithelial cells in wound healing conditions compared to cytoplasmic retention of Sox9 in control tissues (inserts). Nuclear staining: DAPI. (**D**) Relative expression of SOX9 in limbal epithelial cells of wounded and unwounded corneas as determined by quantitative real-time polymerase chain reaction (qRT-PCR) primer assays. Normalized data are expressed as means (2^−∆CT^ × 1,000) ± SD (n = 5) relative to unwounded controls; p = 0.08, unpaired *t*-test.

To delineate the potential role of Sox9 in the maintenance, proliferation and differentiation of LEPC, we first analyzed Sox9 expression in primary human LEPCs cultivated as clones on a growth-arrested 3T3 feeder layer or as monolayers up to two passages (P0-P2) in the absence of feeder cells. Real-time PCR analysis showed that highest mRNA levels of *SOX9* were expressed in feeder-supported clonal cells (Fig. 4A). In feeder-free cultures, expression levels were significantly lower, but did not markedly change during passaging of cells. Immunofluorescent labeling of Sox9 in LEPC clones showed a nuclear staining pattern, with immunopositive cells being located predominantly towards the proliferating periphery of the clones in close association with Ki-67 positive cells (Fig. 4B). These findings indicate increased nuclear expression of Sox9 under culture conditions that promote proliferation of LEPCs.

In view of the co-localization of Sox9 and the proliferation marker Ki-67 *in situ* and *in vitro*, the involvement of Sox9 in corneal epithelial wound healing was assessed using a human corneal organ culture wound healing model. Immunolabeling of Sox9 in cryosections of human donor corneas following epithelial debridement and regeneration (n = 5) showed an increased nuclear staining reaction of Sox9 in both activated limbal and re-grown corneal epithelial cells as well as in keratocytes of the anterior stroma compared to unwounded contralateral control corneas (Fig. 4C). While resting LEPCs of the controls showed cytoplasmic staining for Sox9 as described above, wounding induced re-location of Sox9 to the nucleus. Accordingly, the percentage of epithelial cells showing nuclear Sox9 staining increased from 40.4 ± 7.6% of epithelial cells in controls to 82.5 ± 2.6% of cells in the limbus and from 55.0 ± 4.1% of epithelial cells in controls to 95.8 ± 2.1% of cells in the central cornea upon wound healing. Real-time PCR analysis of limbal epithelial cells after epithelial wounding showed only a moderate, statistically not significant increase in *SOX9* expression levels (1.5-fold) compared to cells from control specimens (Fig. 4D). These findings suggest that when LEPCs are activated to proliferate and differentiate, this occurs concurrently with a change in subcellular localization of Sox9 rather than with an upregulation of *SOX9* expression.

Antibody binding was abolished in negative control experiments using isotype-specific mouse IgG/IgM and rabbit IgG indicating specificity of primary antibodies (Supplementary Fig. 3).

### Functional role of Sox9 expression for limbal epithelial cell function *in vitro*

**Figure 5:**
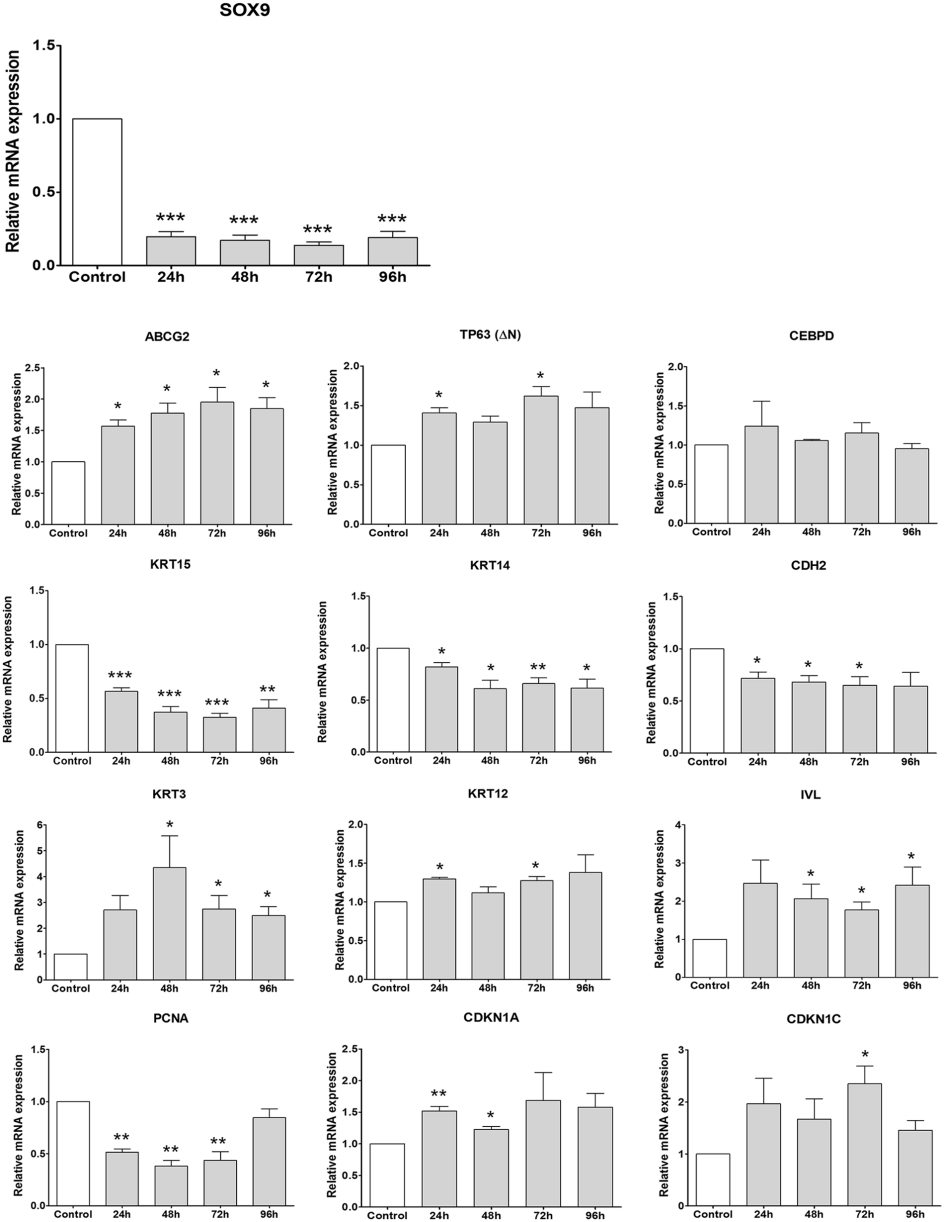
Changes in mRNA expression following knockdown of SOX9 in cultured limbal epithelial cells by RNAi. (**A**) Results of quantitative real-time polymerase chain reaction (qRT-PCR) showing reduction of SOX9 mRNA transcripts in cultured limbal epithelial cells 24–96 hours after transfection with siRNA to SOX9 relative to control cells transfected with scramble siRNA (Control) (n = 6; Mean ± SD). (**B**) Transcriptional changes following knockdown of SOX9 in cultured limbal epithelial cells as determined by qRT-PCR. Significant or no relevant changes were seen in the expression levels of stemness-related genes ABCG2 (ATP Binding Cassette Subfamily G Member 2), TP63 (ΔNp63α) and CEBPD (CCAAT/enhancer-binding protein delta); progenitor cell marker genes KRT15 (keratin 15), KRT14 and CDH2 (N-cadherin); differentiation marker genes KRT3, KRT12 and IVL (involucrin); and proliferation-related genes PCNA (proliferating cell nuclear antigen), CDKN1A (cyclin-dependent kinase inhibitor 1A, p21) and CDKN1C (p57). Normalized data are expressed as means (2^−∆CT^ × 1,000) ± SD (n = 6) relative to scramble siRNA-transfected control cells; *p < 0.05, **p < 0.01, ***p < 0.001, unpaired *t*-test.

TF overexpression in a cell type, which endogenously expresses this gene at relatively high levels, may not lead to gene regulatory changes. Hence, *SOX9* was knocked down in cultured LEPCs by the use of RNA interference (RNAi) to further delineate the potential role of Sox9 in maintenance, proliferation and/or differentiation of LEPCs. At 24 to 96 hours following knockdown of *SOX9* expression in cultured LEPC (n = 6), *SOX9* mRNA levels were reduced by 80–86% compared to scramble siRNA-transfected cells (p < 0.001; Fig. 5A). We then analyzed expression levels of putative stem cell marker genes (*ABCG2, TP63 [ΔN], CEBPD*), progenitor cell marker genes (*KRT15, KRT14, CDH2*), differentiation-related genes (*KRT3, KRT12, IVL*), and genes related to control of proliferation (*PCNA, CDKN1A, CDKN1C, CCND1*). Expression levels of *ABCG2* and *TP63 [ΔN]* were upregulated in cells with reduced expression of *SOX9*, whereas no significant changes were seen in expression of *CEBPD* (Fig. 5B). Moreover, *KRT15, KRT14* and *CDH2* were significantly downregulated, whereas *KRT3*, *KRT12* and *IVL*, a marker of terminal differentiation, were upregulated following knockdown of *SOX9*. The most significant effect could be observed on the expression levels of *KRT15*, which was downregulated up to 3-fold (p < 0.001) in cells transfected with *SOX9*-specific siRNA compared to scramble siRNA-transfected control cells. Finally, we observed a significant downregulation of the proliferation marker *PCNA* together with a moderate upregulation of cyclin-dependent kinase inhibitors *CDKN1A* and *CDKN1C* (Fig. 5B); however, no effect was seen on the expression of *CCND1* (cyclin D1; not shown).

**Figure 6:**
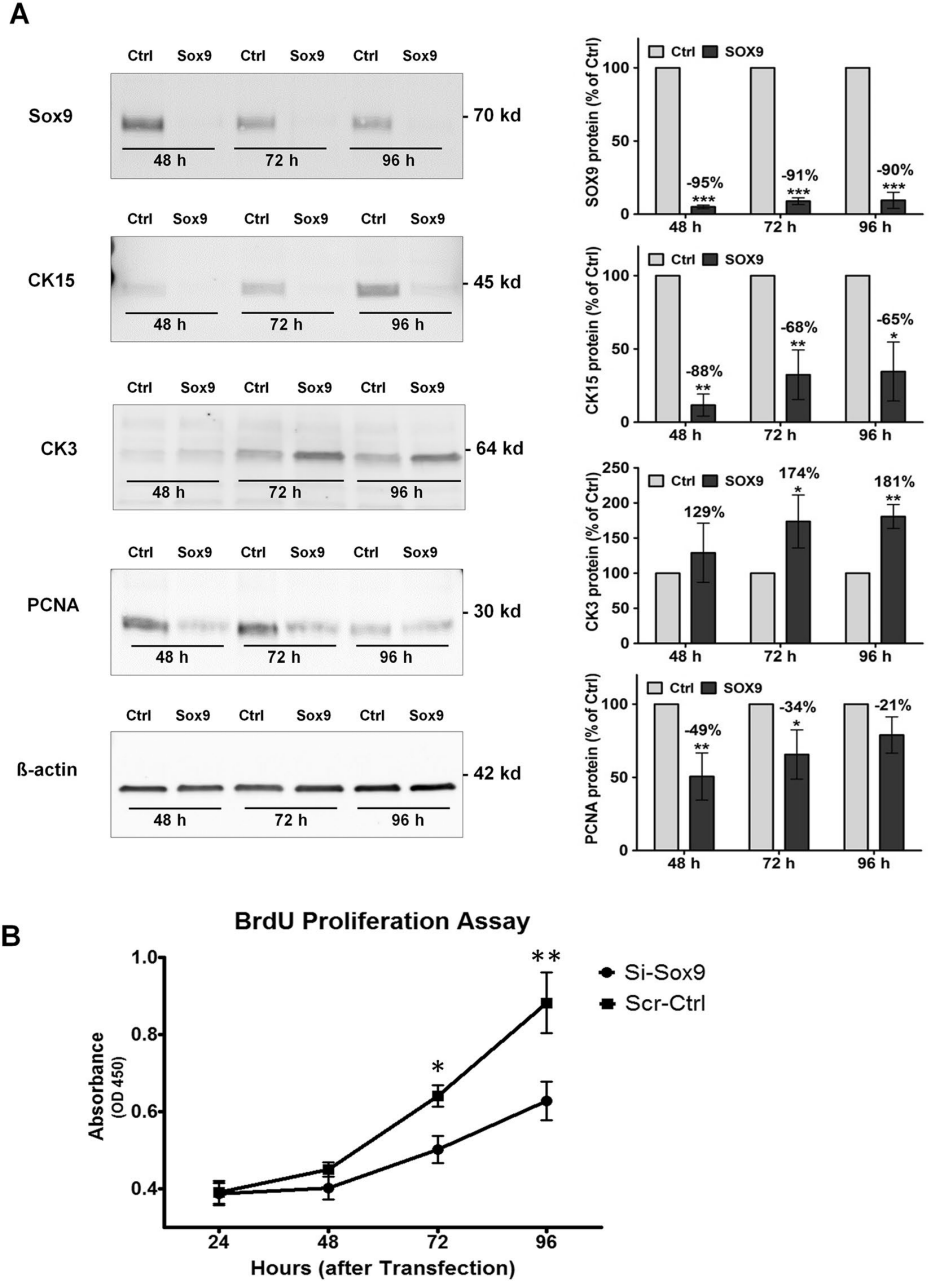
Changes in protein expression and proliferation following knockdown of SOX9 in cultured limbal epithelial cells by RNAi. (**A**) Western blot analysis of limbal epithelial cells transfected with either siRNA that targets SOX9 or non-targeting, scrambled siRNA as a control. Protein expression of Sox9, cytokeratin (CK) 15, CK3 and PCNA was detected with monoclonal antibodies, normalized to the house-keeping gene ß-actin, and expressed as percent of the expression in control cells (Ctrl); (n = 3; mean ± SD); *p < 0.05, **p < 0.005, ***p < 0.0001, unpaired *t*-test. Uncropped versions of Western blots are shown in Supplementary Fig. 4. (**B**) BrdU incorporation (i.e., cell proliferation) was determined by measuring absorbance at 450 nm. Statistically significant differences were observed at 72 (*p = 0.005) and 96 hours (**p = 0.009) between cells transfected with siRNA that targets SOX9 (Si-Sox9) and control cells transfected with scramble siRNA (Scr-Crtl) (n = 3; mean ± SD).

At the protein level, efficient knockdown of Sox9, which appeared as a specific band at 70 kDa, was confirmed by Western blot analysis up to 96 hours post-transfection (Fig. 6A, Supplementary Fig. 4). In accordance with qRT-PCR results, significantly reduced protein levels of keratin 15 and increased protein levels of keratin 3 were confirmed in cultured LEPC following knockdown of *SOX9* (Fig. 6A, Supplementary Fig. 4). In addition, PCNA was also downregulated in LEPCs following knockdown of *SOX9*. Accordingly, proliferation rates analyzed by BrdU incorporation decreased following knockdown of *SOX9*, in comparison to cells transfected with scramble siRNA. These differences became statistically significant after 72 and 96 hours (p < 0.01) (Fig. 6B).

These findings suggest that Sox9 transcriptionally represses genes that are expressed in stem cells but also in terminally differentiated cells, and induces genes that are expressed in proliferating progenitor cells, i.e., transient amplifying cells. Thus, Sox9 appears to regulate cell proliferation and lineage specification of LEPCs without inducing terminal differentiation.

### Interactions between Sox9 and Wnt/ß-catenin signaling

**Figure 7:**
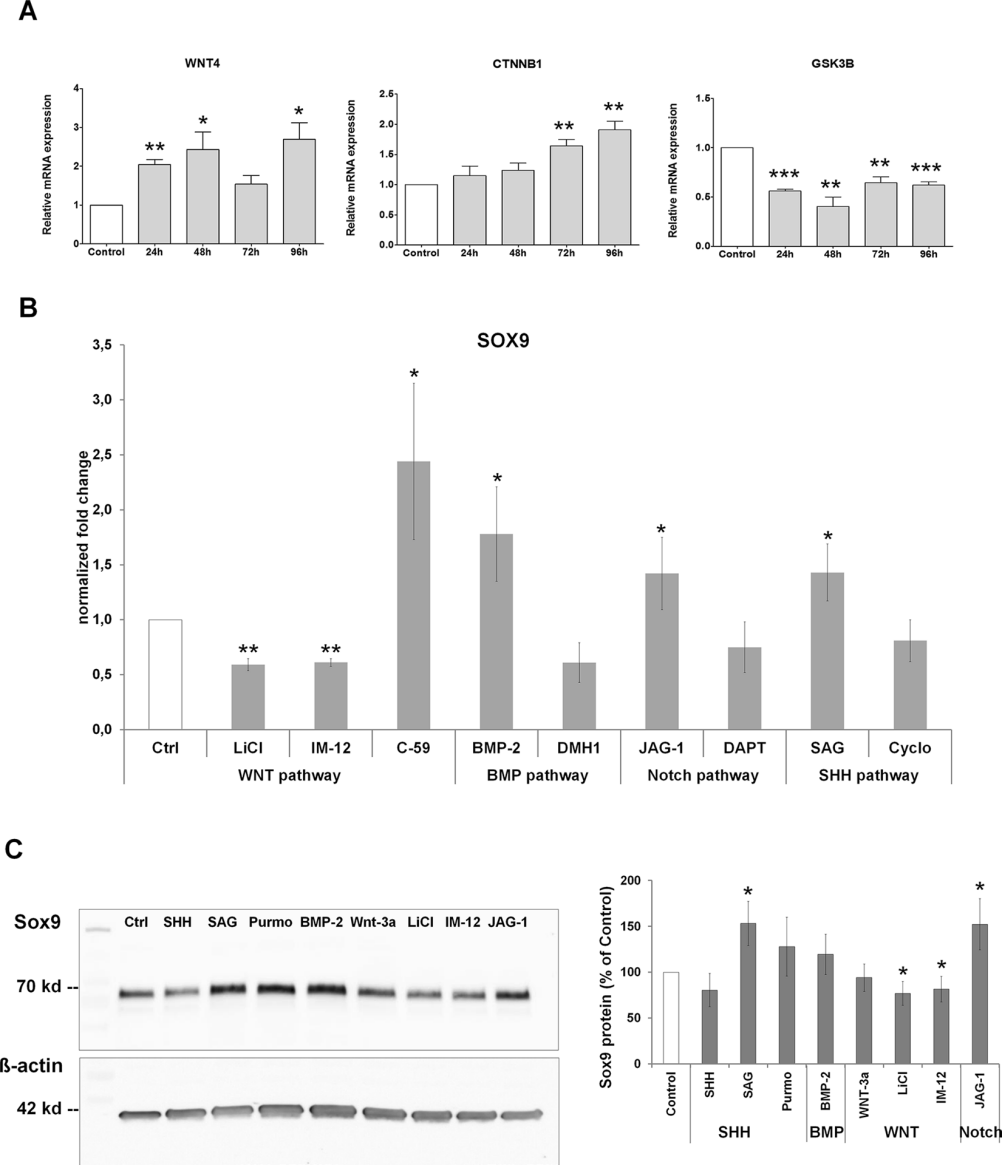
Interactions between Sox9 and cell signaling pathways. (**A**) Changes in mRNA expression of genes centrally involved in Wnt/ß-catenin signaling, i.e., WNT4 (Wnt-4), CTNNB1 (ß-catenin) and GSK3B (glycogen synthase kinase 3 beta), following knockdown of SOX9 in cultured limbal epithelial cells by RNAi relative to mock-transfected control cells. Normalized data are expressed as means (2^−∆CT^ × 1,000) ± SD (n = 3); *p < 0.05, **p < 0.01, ***p < 0.001, unpaired *t*-test. (**B**) Changes in SOX9 mRNA expression following exposure of cultured limbal epithelial cells with signaling activators lithium chloride (LiCl), IM-12, BMP-2, JAG-1 and SAG as well as signaling inhibitors C-59, DMH1, DAPT and Cyclopamine (Cyclo) for 24 hours relative to vehicle-treated control cells (Ctrl). Normalized data are expressed as means (2^−∆CT^ × 1,000) ± SD (n = 3); *p < 0.05, **p < 0.01, unpaired *t*-test. (**C**) Changes in Sox9 protein expression following exposure of cultured limbal epithelial cells to Hedgehog signaling activators Sonic hedgehog (SHH), SAG and Purmorphamine (Purmo); BMP-2; Wnt signaling activators Wnt-3a, lithium chloride (LiCl) and IM-12; and Notch signaling ligand JAG-1 for 48 hours relative to vehicle-treated control cells (Ctrl). Sox9 protein expression was detected with the monoclonal antibody (clone 3C10), normalized to the house-keeping gene ß-actin, and expressed as percent of the expression in control cells (Ctrl); (n = 3; mean ± SD). Uncropped versions of Western blots are shown in Supplementary Fig. 1.

Wnt/ß-catenin signaling has been suggested to regulate LEPC proliferation without inducing their terminal differentiation^16^. Because the Sox family of TF has emerged as important modulators of canonical Wnt signaling in development and disease^17^, we first analyzed, whether Sox9 transcriptionally regulates effectors of the Wnt/ß-catenin pathway, i.e., Wnt-4^18^, ß-catenin and glycogen synthase kinase (GSK)-3ß, a key negative regulator of Wnt signaling^19^. Following siRNA-mediated knockdown of *SOX9* in primary human LEPCs (n = 3), we observed a partly significant increase in expression levels of *WNT4* and *CTNNB1*, and a highly significant decrease in the expression levels of *GSK3B* up to 96 hours post-transfection compared to scramble siRNA-transfected controls (Fig. 7A). These data suggest an attenuation of Wnt/ß-catenin signaling by Sox9.

Besides transcriptionally regulating Wnt activity, *SOX9*, in turn, may be a primary target of Wnt and/or other signaling pathways, such as bone morphogenetic protein (BMP), Notch, and Sonic hedgehog (Shh) pathways^20–24^, which also have been previously implicated in LEPC homeostasis^25^. To further determine, whether *SOX9* may be regulated by these signaling cascades, we analyzed the effect of respective agonists and antagonists of the Wnt, BMP, Notch and Shh pathways on Sox9 expression in primary human LEPC cultures (n = 3). These experiments showed that *SOX9* mRNA levels were significantly downregulated by the GSK-3ß inhibitors lithium chloride (LiCl) and IM-12, but upregulated by the small molecule Wnt inhibitor C59 after 24 hours of exposure, compared to vehicle-treated control cells (Fig. 7B). In contrast, *SOX9* was moderately upregulated after treatment of LEPC with BMP-2 (bone morphogenetic protein-2), JAG-1 (Jagged-1, Notch ligand) and SAG (Smoothened agonist), indicating its induction by BMP, Notch and Shh signaling pathways. Downregulation of *SOX9* expression by the corresponding pathway inhibitors DMH1 (dorsomorphin homolog 1), DAPT (γ-secretase inhibitor) and cyclopamine was observed but did not reach statistical significance. These data indicate that *SOX9* expression is suppressed by Wnt signaling and stimulated by BMP, Notch and Shh signaling activation.

Western blot analysis (n = 3) confirmed that Sox9 protein was downregulated by the Wnt activators LiCl and IM-12 compared to vehicle-treated control, although recombinant Wnt-3a had only little effect on Sox9 expression (Fig. 7C, Supplementary Fig. 4). Upregulation of Sox9 protein was observed upon treatment with the Shh activators SAG and purmorphamine (Smoothened agonist), BMP-2 and JAG-1, although statistical significance was only reached with SAG and JAG-1. Also, human recombinant Shh had no significant effect.

**Figure 8:**
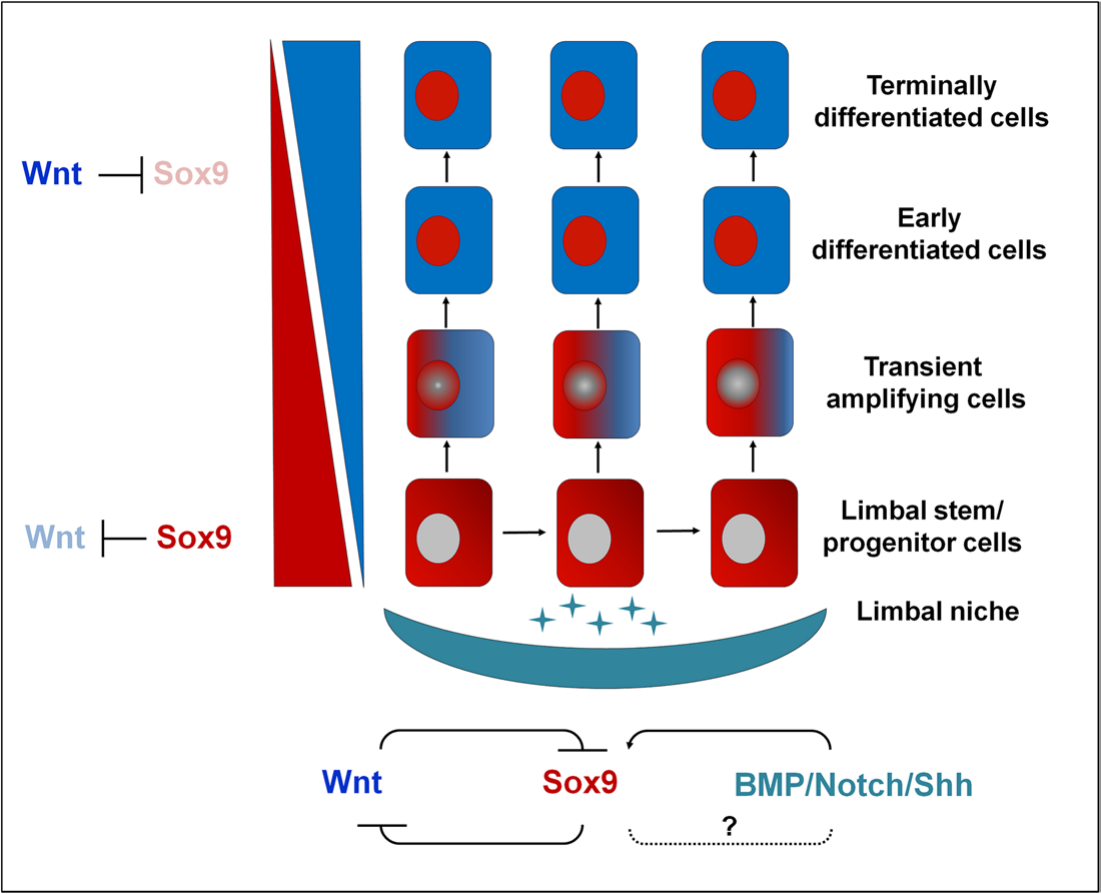
Model illustrating the mutually repressive interaction of Sox9 and Wnt/ß-catenin signaling as well as potentially involved regulatory signaling pathways in the limbal stem cell niche (mod. after Xu, Z. *et al*., Elife 4, e10567 (2015) (https://creativecommons.org/licenses/by/4.0).

Altogether, these *in vitro* experiments suggest that, on the one hand, Sox9 antagonizes Wnt/ß-catenin signaling in LEPCs by means of upregulation of GSK-3ß as part of the ß-catenin destruction complex. On the other hand, Sox9 expression, in turn, is negatively regulated by Wnt/ß-catenin signaling and positively regulated by other cell signaling pathways, including BMP, Notch and Shh, operating in the limbal niche. The mutually repressive interaction between Sox9 and Wnt signaling may cooperate in regulating LEPC function and fate (Fig. 8).

## Discussion

To better understand cellular behaviour in the context of heterogeneous tissues, LCM offers the technological means to harvest distinct cell populations directly from their complex tissue microenvironment^26^. In a previous study, we have shown that this technique yields valid gene expression data from distinct epithelial cell populations at the ocular surface in strict accordance with appropriate quality control measures^15^. Here, this approach has allowed us to detect overexpression of a small number of TF genes in limbal epithelial stem/progenitor cells compared with basal corneal epithelial cells. Strong preferential expression in LEPC clusters was consistently detected for *DACH1, HOXA11*, and *PPARG* in all samples analyzed. These TF have previously been suggested as important regulators of cell fate determination and proliferation^27^, stem cell maintenance and self-renewal^28^ and differentiation^29^. Because of its established role for ocular surface physiology^30^, more detailed analysis of *PPARG* (peroxisome proliferator-activated receptor gamma) was therefore transferred into a separate study. Moreover, further analysis of *DACH1* (Dachshund homolog 1) and Hox (homeobox) gene expression and function may aid in the molecular dissection of limbal stem cell regulation.

In this study, further analysis concentrated, however, on the Sox family of TFs, because expression data suggested most pronounced differential expression for Sox family member *SOX9*, which has been shown to be of high relevance for stem cell function^31^. Also, family member *SOX2* is known to be of high relevance in the context of adult stem cells and reprogramming^32^. In our epithelial samples, however, *SOX2* expression was not detected. Instead, real-time PCR expression data indicated that all members of the SoxE group and the SoxF group show preferential expression in limbal progenitor cells. The SoxF group has assigned roles in endoderm formation, vascular and hair development, but its expression or function in stem cell compartments remain largely undefined^33^. In our hands, immunofluorescent staining did not detect SoxF proteins at the human corneoscleral limbus in significant amounts, with the possible exception of Sox17, which labelled suprabasal nuclei in the corneo-limbal epithelium. It was reported that in gut epithelium, Sox17 antagonizes the proliferative effect of Wnt signals by increasing degradation of the β-catenin/TCF complex^34^. The notion that Sox17 may contribute to maintaining the balance between Wnt-mediated activation and stem cell quiescence in corneal epithelial homeostasis warrants further research. But at present, the roles of Sox7, Sox17 and Sox18 in LEPCs remain elusive, not least because of the unavailability of efficient and specific antibodies.

Unlike SoxF, a plethora of reports indicate relevance of members of the SoxE group for stem cell function. Our qPCR and immunofluorescence data confirmed preferential limbal localization of Sox8, Sox9 and Sox10. Results from co-labeling experiments are in agreement with studies in mice that have suggested that most Sox8-expressing cells are also positive for Sox9^35^. Thus, previous studies have proposed functional redundancy within the SoxE group, with loss of Sox8 being compensated for by Sox9 or Sox10 but not vice-versa^36^. Among other mechanisms, it has been suggested that differences in levels of expression could at least partly explain these findings. Indeed, relative expression levels of Sox8 in limbal cells were much lower than levels of Sox9 and Sox10. Also, we observed that histologically, Sox8-deficient mice showed no overt phenotype at the corneal surface at the age of six months (data not shown), supporting the notion of Sox8 redundancy.

Other authors had reported that Sox10 is expressed in human adult limbal epithelium^18,37^. However, microarray data from these studies was not validated *in situ*. Our immunofluorescent staining of limbal sections demonstrated that Sox10 protein is exclusively expressed in Melan-A positive melanocytes within the basal epithelial cell layer at the limbus. In postnatal mice, melanocytes maintain and are maintained by expression of high levels of Sox10, while Sox10 activity in melanocyte stem cells is decreased^38^. It is commonly accepted that melanocytes at the limbus serve to shield LEPCs from ultraviolet radiation. However, further involvement of these cells in limbal stem cell biology has not yet been thoroughly investigated, but a recent report suggests human limbal melanocytes may have additional functions in the maintenance of LEPCs^39^.

Given the low expression and assumed redundancy of Sox8 and the possible restriction of Sox10 to limbal melanocytes, Sox9 was selected for further analyses because it has been shown to regulate stem cell functions in several stem cell compartments, including those of the retina, brain subventricular zone, hair follicles, skin, intestine, liver, pancreas, mammary gland and cartilage^31,33,40–43^. By alternatively regulating stem cell maintenance, lineage specification, proliferation and differentiation in these various compartments, Sox9 has been implicated in the governance of multiple adult stem cell pools and tissue regeneration. Sox9 has also been previously identified in limbal epithelial cells by microarray analysis of scraped epithelial samples^44^ and transcriptome analysis of microdissected limbal epithelial crypts^37^ as well as in cultivated human limbal epithelial keratinocytes^45^, but was not further analysed *in situ*. In addition, Sox9 has been identified as a marker of slow-cycling corneal epithelial stem cells in mouse eyes^46,47^. In extending this anecdotal evidence for a role of Sox9 in the human limbal stem cell niche, the present study demonstrated a striking differential sub-cellular localization of Sox9 in basal LEPC clusters and their progeny: Whereas LEPCs showed mainly cytoplasmic staining for Sox9, indicative of protein synthesis, suprabasal limbal and corneal epithelial cells showed exclusively nuclear localization suggestive of TF activity. Controlled access of proteins to the nucleus is known to be a key driver of developmental switches and programmed cell differentiation^48^. In addition to pre- and post-transcriptional regulation, nucleocytoplasmic shuttling has been identified as an alternative mechanism to dynamically regulate the activity of TFs of the SoxE group, and particularly that of Sox9, in response to signaling molecules^49^. Two conserved nuclear localization signals have been characterized within the DNA-binding high mobility group (HMG) domain of Sox proteins^48,49^. These interact with calcium-activated calmodulin to increase nuclear import and subsequent transcriptional activity. Altogether, these observations suggest that abundant cytosolic expression of Sox9 characterizes LEPC maintenance and quiescence, and that the translocation from its site of synthesis in the cytoplasm to its site of action in the nucleus parallels proliferation and early differentiation of their progeny, i.e., transient amplifying cells^50^. Increase in nuclear expression of Sox9 during LEPC *ex vivo* expansion and corneal epithelial wound healing further supports the notion, that this TF may be functionally involved in transcriptional programs controlling LEPC proliferation and early differentiation.

To corroborate this notion, we carried out RNAi experiments to study the effects of *SOX9* knockdown in primary human LEPCs *in vitro*. We observed both a significant upregulation of putative stem cell markers, such as *ABCG2*, and terminal differentiation markers, such as *KRT3 and IVL*, together with a downregulation of progenitor cell markers, particularly *KRT15*, on the mRNA and protein level. Furthermore, the proliferation marker *PCNA* was significantly downregulated in LEPCs after *SOX9* knockdown, consistent with a decreased rate of cellular proliferation. In contrast, the negative cell cycle regulators *CDKN1A* (cyclin-dependent kinase inhibitor p21) and *CDKN1C* (p57) were moderately upregulated upon *SOX9* silencing possibly mediating an inhibitory effect on proliferation. Taken together, these findings further support the concept that Sox9 regulates proliferation and early differentiation of LEPCs and their transient amplifying progenitors, without inducing their terminal differentiation. However, potentially integral to the maintenance of properly differentiated cells, Sox9 remains localized to the nucleus of differentiated cells throughout the corneal epithelium. These data comply with reports from other stem cell compartments^21,41^ and with the general concept, that SoxB1 genes (such as *SOX2*) control stem cell quiescence and maintenance, while SoxE genes work downstream to control proliferation, lineage specification and early differentiation^51^.

Importantly, it has been suggested that Sox9 may regulate stem cell function through transcriptional modulation of genes involved in Wnt signaling^52,53^. While Sox genes clearly have Wnt independent roles, there are numerous reports in the literature, where Sox and Wnt are implicated in the same biological processes. In line with this concept, we showed here, that siRNA-mediated knockdown of *SOX9* induced upregulation of the Wnt ligand *WNT4* (Wnt-4) and *CTNNB1* (ß-catenin), the key downstream effector of the canonical Wnt pathway^54^. In contrast, *GSK3B* (glycogen synthase kinase 3 beta), which negatively regulates Wnt signaling by phosphorylating and inactivating ß-catenin, was significantly downregulated following *SOX9* silencing in cultured LEPC. These data indicates that high expression levels of Sox9 in LEPCs attenuate Wnt/ß-catenin signaling in the limbal stem cell niche by increasing degradation of the ß-catenin complex. It is consistent with previous studies showing that Wnt signaling appears not activated in LEPCs *in vivo*^55^, and that Wnt signaling must be repressed for maintaining a stem cell phenotype and for proper development, differentiation and stratification of the corneal epithelium^45,56,57^. Others, however, suggested that activation of Wnt signaling is required for LEPC proliferation and differentiation during corneal epithelial homeostasis^16,18,58,59^.

Besides transcriptionally regulating the Wnt pathway, Sox9 may act as primary target downstream of various signaling pathways including Wnt, BMP, Notch and Shh pathways^20–24^. Here, we show that Wnt/ß-catenin signaling suppressed Sox9 expression, whereas agonists/activators of the BMP, Notch and Shh pathways induced its expression in primary human LEPCs *in vitro*. These observations are also in line with a recent report that activation of Wnt signaling leads to downregulation of *SOX9* in cultured human limbal epithelial cells and that this is associated with a reduction of proliferative capacity in these cells^45^. In a similar fashion, the hair follicle niche location is defined by attenuated Wnt/ß-catenin signalling, which is a prerequisite for stem cell specification because it suppresses Sox9, which is required for stem cell maintenance^60^.

It may be interesting to note that a similar expression pattern to that of Sox9, i.e., cytoplasmic localization in LEPCs and nuclear localization in suprabasal limbal and corneal epithelial cells, has been reported for the TF Yap (yes-associated protein), which is involved in cell mechanotransduction and acts as a major regulator of cell growth and differentiation downstream of the Hippo signalling pathway^61,62^. It has, therefore, been suggested that Yap, dependent on its subcellular localization, might represent a possible master regulator of corneal epithelial cell proliferation, migration and differentiation in response to biophysical cues. Its effects on cell functions appear to be supported by interaction with Wnt/ß-catenin signalling^63^. Moreover, Yap has been shown to regulate *SOX9* transcription through direct promoter binding, thereby functioning as a transcriptional activator or repressor in a cell- and tissue-specific manner^64,65^. Although Yap was not included in our initial TF profiler PCR array, these studies suggest that the Yap-Sox9 axis in cross-talk with the Wnt/ß-catenin signalling pathway may be a key player in corneal epithelial homeostasis.

## Summary and Conclusion

In summary, this study identified Sox9 as a significant marker of limbal stem/progenitor cells, as reflected by both high expression levels and cytoplasmic localization of this TF in LEPCs. Expression of Sox9 in LEPCs may be induced by various signaling pathways, including BMP, Notch and Shh, operating in the limbal niche. Cytoplasmic retention of Sox9 in LEPCs seems to be associated with stem cell quiescence and maintenance. Controlled translocation of Sox9 from its site of synthesis, the cytoplasm, to its site of action, the nucleus, may trigger transition of LEPCs into proliferating, transient amplifying cells and their differentiation along the correct lineage to attain regenerative potential. The signals and mechanisms underlying this transition from an inactive to an active state are currently not known, but might involve receptor signaling by growth factors and cytokines, such as TGF-ß1^49,66^. Our results further suggest that Sox9 and Wnt/ß-catenin signaling cooperate in mutually repressive interactions to attenuate canonical Wnt signaling in the limbal niche and to regulate LEPC function and fate. However, how Sox9 and Wnt signaling cooperate in achieving a balance between stem cell quiescence, self-renewal, fate decision, and Wnt-mediated activation of proliferation and differentiation, warrants further investigation.

Evidence that Sox9 can potentially be used in the context of cell reprogramming comes from a study reporting that co-expression of Sox9 and Slug in differentiated murine luminal cells of mammary duct produced induced multipotent cells with mammary gland reconstituting potential^43^. Further evidence suggesting that this TF can potentially be used in the context of cell reprogramming for corneal epithelial regeneration is provided by a study reporting that Sox9, together with Pax6, Klf4 and Ovo-like 2, is required for the activation of corneal epithelial cell-specific genes in cultured human fibroblasts^67^. To move further towards the use of Sox9 in regenerative procedures for corneal epithelium, its signaling interactions, co-factors and mechanisms of nucleocytoplasmic shuttling require further studies. Nucleocytoplasmic shuttling can be modulated experimentally, for instance through inhibition of Sox9 nuclear export by leptomycin B^49^. Manipulation of nuclear import/export and sub-cellular localization of Sox9 may thus constitute a viable means to further assess the functional role of Sox9 in LEPCs, to transdifferentiate non-ocular cells into a corneal epithelial phenotype, and to control LEPC maintenance, proliferation and differentiation during *ex vivo* expansion for ocular surface regeneration.

## Materials and Methods

### Human tissues and study approval

Human donor corneas not suitable for transplantation with appropriate research consent were procured by the Erlangen Cornea Bank. Informed consent to corneal tissue donation was obtained from the donors or their relatives. Experiments using human tissue samples were approved by the Institutional Review Board of the Medical Faculty of the University of Erlangen-Nürnberg (No. 4218-CH) and adhered to the tenets of the Declaration of Helsinki.

### Laser capture microdissection (LCM) and amplification of RNA

LCM and amplification of RNA was performed as previously described^15^. Briefly, corneal specimens destined for LCM were obtained from five donors (mean age, 69.6 ± 10.4 years) within 15 hours after death. After labeling of the superior, inferior, nasal, and temporal quadrants of donor globes, tissue sectors were embedded in optimal cutting temperature (OCT) compound (Tissue-Tek, Sakura Finetek Europe) and snap frozen in liquid nitrogen. Roughly 100 serial cryosections of 12 μm thickness were obtained under RNAse-free conditions from the superior or inferior quadrants of each donor eye, placed onto UV-irradiated (3000 mJ/cm^2^) PEN (polyethylene naphtalate) Membrane Slides (Carl Zeiss Microscopy, Göttingen, Germany), and stained with 1% cresyl violet. The PALM MicroBeam IV system (Carl Zeiss Microscopy) was used to isolate clusters of basal limbal epithelial progenitor cells (LEPC) and basal epithelial cells from central cornea (BCEC).

RNA isolation from these specimens was achieved using the RNeasy Micro Kit (Qiagen, Hilden, Germany) including an on-column DNase digestion step according to the manufacturer’s instructions. Quality control was performed on a 2100 Agilent Bioanalyzer using the RNA 6000 Pico Kit (Agilent Technologies, Santa Clara, CA). Samples with an RNA concentration of 650–2,000 pg/µl and a RIN (RNA integrity number) of ≥7.0 were used for amplification. Following RNA-amplification using the MessageAmp II aRNA Amplification Kit (Life Technologies GmbH, Darmstadt, Germany) according to the manufacturer’s protocol, aRNA (amplified RNA) concentration was measured on a Nanodrop ND1000 spectrophotometer (Thermo Scientific, Wilmington, DE) and quality control was again performed using Agilent technology.

### Real time RT-PCR

Differential gene expression analysis was performed using the RT^2^ Profiler PCR Array Human Stem Cell Transcription Factors (Qiagen). First strand cDNA synthesis was performed with 5 µg of high-quality aRNA using the RT^2^ First Strand Kit (Qiagen) according to the manufacturer’s instructions. qPCR was carried out using the CFX Connect Real Time System and software (BioRad, Munich, Germany) and the RT^2^ SYBR Green qPCR master mix (Qiagen) according to the manufacturer’s protocol. Data were analyzed using the RT^2^ Profiler PCR array data analysis tool version 4.0 (Qiagen). PCRs were run using the following program: 95 °C for 10 minutes, followed by 40 cycles of 95 °C for 15 seconds and 60 °C for 60 seconds. Supplementary Table 1 shows Reference Sequence numbers (RefSeq) of the respective transcripts as well as symbols and names of all 84 genes examined.

Since RNA-amplification may produce 5′-truncated cDNA^68^, array results were confirmed using custom-designed quantitative real-time PCR (qRT-PCR) assays. First-strand cDNA synthesis was performed using 5 µg of aRNA and Superscript II reverse transcriptase (Invitrogen, Karlsruhe, Germany) as previously described^15^, and PCR reactions were run in triplicate with Universal ProbeLibrary probes (Roche Diagnostics) and primers targeting the 3’-region. The Roche Universal ProbeLibrary Assay Design Center was used to determine primer sequences and probes (Supplementary Table 2 and Supplementary Table 3). The following real-time PCR-program was used: 95 °C for 10 minutes, followed by 40 cycles of 95 °C for 10 seconds and 60 °C for 30 seconds. For normalisation of gene expression, ratios relative to the housekeeping gene *GAPDH* were calculated by the comparative *C*_T_ method (ΔΔ*C*_T_).

### Immunohistochemistry

Corneoscleral tissue samples obtained from 10 normal human donor eyes (mean age, 78.7 ± 9.7 years) were embedded in optimal cutting temperature (OCT) compound and snap frozen in isopentane-cooled liquid nitrogen. Cryosections of 4 μm thickness were cut from the superior or inferior quadrants, fixed in cold acetone or 4% paraformaldehyde for 10 minutes, washed in phosphate balanced saline (PBS), and permeabilised using 0.1% Triton X-100 in PBS for 10 minutes. After blocking with 10% normal goat serum, sections were incubated over night at 4 °C in primary antibodies (Supplementary Table 4) diluted in PBS. Antibody binding was detected by Alexa Fluor^®^ 488- or 555-conjugated secondary antibodies (Life Technologies). Nuclear counterstaining was achieved using 4′,6′-diamino-2-phenylindole (DAPI; Sigma-Aldrich, St. Louis, MO). Slides were washed and coverslipped with Vectashield mounting medium (Vector Laboratories) prior to evaluation on a fluorescence microscope (BX51, Olympus, Hamburg, Germany) or a laser scanning confocal microscope (LSM 780; Carl Zeiss Microscopy). In negative control experiments, the primary antibodies were replaced by equimolar concentrations of isotype-specific mouse and rabbit immunoglobulins (Supplementary Table 4) or irrelevant isotypic primary antibodies.

### Organ culture wound healing

Pairs of whole donor corneas (n = 5) not suitable for transplantation with appropriate research consent were used in *in vitro* wound healing experiments. A central epithelial debridement zone with a diameter of 6 mm was created in one cornea using a hockey knife (Geuder, Heidelberg, Germany). The contralateral donor eye served as untreated control. Corneas were incubated using standard European eye bank conditions for 72 hours. Following incubation, corneas were cut into two halves: one half was processed for immunohistochemistry and the other half was processed for RNA isolation of corneal epithelium as described above.

### Limbal epithelial cell culture

Specimens destined for limbal epithelial cell cultures were prepared according to national and European regulations for eye banking and in agreement with national guidelines established by the German Medical Association. Following clinical use for corneal endothelial transplantation, corneal buttons obtained from 20 donors (mean age 66.7 ± 9.2 years) with appropriate research consent were used for limbal epithelial cell cultivation. LEPC clusters were isolated as previously described^50^. Briefly, the tissues were cut into 12 one-clock-hour sectors, from which limbal segments were obtained by incisions made at 1 mm before and beyond the anatomical limbus. Each limbal segment was enzymatically digested with 2 mg/mL collagenase A (Roche Diagnostics) at 37 °C for 16 hours and cell clusters containing LEPC were isolated from single cells by using reversible cell strainers with a pore size of 20 µm (Stem Cell Technologies, Köln, Germany). Isolated cell clusters were further dissociated into single cells by digestion with 0.05% trypsin and 0.02% EDTA (Pan Biotech, Aidenbach, Germany) at 37 °C for 10–15 min. Single cell suspensions were seeded into T75 flasks (Corning, Tewksbury, MA) in Keratinocyte serum free medium (KSFM) supplemented with bovine pituitary extract, epidermal growth factor (Life Technologies) and 1× penicillin-streptomycin-amphotericin B mix (Pan Biotech) to enrich epithelial cell population and the flasks were incubated at 37 °C under 5% CO_2_ and 95% humidity. For clonal expansion of LEPC, single cell suspensions were seeded at a density of 1 × 10^3^ cells/cm^2^ on a feeder layer of growth-arrested murine 3T3 fibroblasts in 6 well-plates and cultured in either KSFM or equal parts of Dulbecco’s modified Eagle’s medium and Ham’s F12 medium (DMEM/F12; Pan Biotech) supplemented with 10% fetal calf serum, 1% Human Corneal Growth Supplement (Thermo Scientific), 5 ng/ml human epidermal growth factor (Invitrogen), and 5 µg/ml gentamycin. The media was changed every second day.

For activation of Wnt signaling, primary human LEPC (P1) were exposed to the Wnt ligand Wnt-3a (100 ng/ml; R&D Systems), lithium chloride (LiCl; 5 µM; Sigma-Aldrich) or a glycogen synthase kinase-3ß (GSK-3ß) inhibitor (IM-12; 5 µM; Sigma-Aldrich); for inhibition of Wnt signaling, cells were treated with the Wnt antagonist C59 (5 µM; Abcam). Recombinant human BMP-2 (50 ng/ml; R&D Systems), recombinant human Jagged-1 (JAG-1, active fragment; 50 µM; Anaspec), recombinant human Sonic hedgehog (SHH, N-Terminus; 2.5 µg/ml; R&D Systems), and the Smoothened agonists Purmorphamine (50 µM; Calbiochem) and SAG (10 µM; Calbiochem) were used to activate the BMP, Notch and Hedgehog signaling pathways, respectively. The BMP antagonist DMH1 (30 µM; Sigma-Aldrich), the γ-secretase inhibitor DAPT (20 µM; Sigma-Aldrich) and the steroidal alkaloid Cyclopamine (10 µM; Abcam) were used to inhibit BMP, Notch and Hedgehog signaling activities, respectively. Doses of agents were administered to cells according to recommendations by pertinent publications and manufacturers’ instructions. Cells treated with solvent, e.g., dimethyl sulfoxide (DMSO) or PBS alone (vehicle), served as controls. After 24 or 48 hours of exposure, cells were processed for real time RT-PCR or Western blot analysis, respectively.

### siRNA silencing

Primary limbal epithelial cells (P1) were transiently transfected with specific siRNA (ON-TARGETplus SMARTpool; GE Healthcare Dharmacon, Freiburg, Germany) for *SOX9* (600 pmol) by electroporation using the Nucleofector II transfection device (Lonza, Köln, Germany) and the Amaxa Cell Line Nucleofector Kit V (Lonza). Transfections with scrambled siRNA (ON-TARGETplus Non-targeting pool, GE Healthcare Dharmacon) served as controls. Transfected cells were seeded into 6-well plates in duplicate and harvested at 24, 48, 72 and 96 hours post-transfection for real-time PCR analysis.

### Proliferation assay

The effect of *SOX9* knockdown on LEPC proliferation was quantified using the Cell Proliferation ELISA BrdU Colorimetric Assay Kit (Roche Diagnostics). Transfected cells were seeded into 24-well plates at a density of 3 × 10^5^ cells/well, cultured for 48, 72 and 96 hours, and labeled with 10 µM BrdU according to the manufacturer’s instructions. Absorbance was measured at 450 nm using a spectrophotometer (Multiskan Spectrum; SLT Labinstruments), and fold change values were calculated as described above.

### Western blot analysis

Total protein was isolated from cultured cells using RIPA buffer (Radioimmunoprecipitation assay buffer; Sigma-Aldrich). Protein concentration was measured using the Micro BCA Protein Assay kit (Thermo Fisher Scientific). 15 μg of total protein was separated by SDS-PAGE under reducing conditions using Mini-PROTEAN TGX Stain-Free Precast Gels (Bio-Rad). It was transferred onto nitrocellulose membranes with a semidry blotting unit (Trans-Blot Turbo, Bio-Rad). Membranes were blocked with SuperBlock T20 Blocking Buffer (Thermo Fisher Scientific) for 1 hour and incubated overnight using monoclonal mouse antibodies against Sox9 (1:5000; clone 3C10; BioRad), Cytokeratin 3/76 (1:5000; clone AE5; Millipore), Cytokeratin 15 (1:1000; clone EPR1614Y; Abcam) and PCNA (1:5000; clone PC10; Abcam). Equal loading of samples was verified with anti-β-actin antibodies (1:5000; clone AC-15; Sigma). In negative control experiments, the primary antibody was replaced by PBS. Immunodetection was performed with horseradish peroxidase-conjugated secondary antibodies (Biolegend) diluted 1:20.000 and the Super Signal West Femto ECL kit (Thermo Fisher Scientific).Specific protein bands were quantitatively analyzed with the LAS-3000 (Fujifilm, Düsseldorf, Germany) chemiluminescence detection system and software (Multi Gauge V1.1, Fujifilm). For normalization of protein expression levels, protein ratios relative to the house-keeping gene β-actin were calculated. Data represents at least three biological replicates.

### Statistical analysis

Statistical analyses were performed using the SPSS v.19 software (IBM, Ehningen, Germany). Data are expressed as mean ± standard deviation from individual experiments. A two-tailed unpaired t-test was performed to assess statistical significance. A *p* value of < 0.05 was considered statistically significant.

### Data availability

Any additional data beyond those included in the main text that support the findings of this study are also available from the corresponding author upon request.

## Electronic supplementary material

Supplementary information
