# Supplementary material for: Transcription factor profiling identifies Sox9 as regulator of proliferation and differentiation in corneal epithelial stem/progenitor cells
Source: Sci Rep. 2018 Jul 6;8:10268. doi: 10.1038/s41598-018-28596-3 (PMC6035181; doi:10.1038/s41598-018-28596-3)

Transcription factor profiling identifies Sox9 as regulator of proliferation and differentiation in corneal epithelial stem/progenitor cells

Johannes Menzel-Severing, Matthias Zenkel, Naresh Polisetti, Elisabeth Sock, Michael Wegner, Friedrich E. Kruse, Ursula Schlötzer-Schrehardt

Supplementary Table 1: Full listing of transcription factor gene assays contained in the RT^2 Profiler qPCR arrays used for analysis of LCM specimens. Product details are available at http://www.sabiosciences.com/rt_pcr_product/HTML/PAHS-501Z.html.

RefSeq	Gene symbol	Gene name
NM_001265	CDX2	Caudal type homeobox 2
NM_004392	DACH1	Dachshund homolog 1
NM_178120	DLX1	Distal-less homeobox 1
NM_004405	DLX2	Distal-less homeobox 2
NM_006892	DNMT3B	DNA (cytosine-5-)-methyltransferase 3 beta
NM_004430	EGR3	Early growth response 3
NM_000125	ESR1	Estrogen receptor 1
NM_004456	EZH2	Enhancer of zeste homolog 2
NM_004496	FOXA1	Forkhead box A1
NM_021784	FOXA2	Forkhead box A2
NM_032682	FOXP1	Forkhead box P1
NM_014491	FOXP2	Forkhead box P2
NM_014009	FOXP3	Forkhead box P3
NM_002049	GATA1	GATA binding protein 1
NM_005257	GATA6	GATA binding protein 6
NM_005270	GLI2	GLI family zinc finger 2
NM_004821	HAND1	Heart and neural crest derivatives expressed 1
NM_018951	HOXA10	Homeobox A10
NM_005523	HOXA11	Homeobox A11
NM_006735	HOXA2	Homeobox A2
NM_030661	HOXA3	Homeobox A3
NM_006896	HOXA7	Homeobox A7
NM_152739	HOXA9	Homeobox A9
NM_002144	HOXB1	Homeobox B1
NM_006361	HOXB13	Homeobox B13
NM_002146	HOXB3	Homeobox B3
NM_002147	HOXB5	Homeobox B5
NM_024016	HOXB8	Homeobox B8
NM_017409	HOXC10	Homeobox C10
NM_173860	HOXC12	Homeobox C12
NM_153633	HOXC4	Homeobox C4
NM_018953	HOXC5	Homeobox C5
NM_004503	HOXC6	Homeobox C6
NM_006897	HOXC9	Homeobox C9
NM_024501	HOXD1	Homeobox D1
NM_002148	HOXD10	Homeobox D10
NM_014621	HOXD4	Homeobox D4
NM_000872	HTR7	5-hydroxytryptamine receptor 7
NM_016358	IRX4	Iroquois homeobox 4
NM_002202	ISL1	ISL LIM homeobox 1

Supplementary Table 1 (contd.)

NM_002228	JUN	Jun proto-oncogene
NM_016270	KLF2	Kruppel-like factor 2
NM_004235	KLF4	Kruppel-like factor 4
NM_001004317	LIN28B	Lin-28 homolog B
NM_002316	LMX1B	LIM homeobox transcription factor 1, beta
NM_002449	MSX2	Msh homeobox 2
NM_002467	MYC	V-myc myelocytomatosis viral oncogene homolog
NM_024865	NANOG	Nanog homeobox
NM_002500	NEUROD1	Neurogenic differentiation 1
NM_172390	NFATC1	Nuclear factor of activated T-cells
NM_002509	NKX2-2	NK2 homeobox 2
NM_024408	NOTCH2	Notch 2
NM_021005	NR2F2	Nuclear receptor subfamily 2, group F, member 2
NM_005806	OLIG2	Oligodendrocyte lineage transcription factor 2
NM_006192	PAX1	Paired box 1
NM_016734	PAX5	Paired box 5
NM_000280	PAX6	Paired box 6
NM_006194	PAX9	Paired box 9
NM_182649	PCNA	Proliferating cell nuclear antigen
NM_000325	PITX2	Paired-like homeodomain 2
NM_005029	PITX3	Paired-like homeodomain 3
NM_006237	POU4F1	POU class 4 homeobox 1
NM_004575	POU4F2	POU class 4 homeobox 2
NM_002701	POU5F1	POU class 5 homeobox 1
NM_015869	PPARG	Peroxisome proliferator-activated receptor gamma
NM_000321	RB1	Retinoblastoma 1
NM_001754	RUNX1	Runt-related transcription factor 1
NM_016932	SIX2	SIX homeobox 2
NM_005901	SMAD2	SMAD family member 2
NM_003106	SOX2	SRY (sex determining region Y)-box 2
NM_033326	SOX6	SRY (sex determining region Y)-box 6
NM_000346	SOX9	SRY (sex determining region Y)-box 9
NM_138473	SP1	Sp1 transcription factor
NM_007315	STAT1	Signal transducer and activator of transcription 1
NM_003150	STAT3	Signal transducer and activator of transcription 3
NM_181486	TBX5	T-box 5
NM_003212	TDGF1	Teratocarcinoma-derived growth factor 1
NM_198253	TERT	Telomerase reverse transcriptase
NM_021025	TLX3	T-cell leukemia homeobox 3
NM_000376	VDR	Vitamin D receptor
NM_000553	WRN	Werner syndrome, RecQ helicase-like
NM_000378	WT1	Wilms tumor 1
NM_012082	ZFPM2	Zinc finger protein, multitype 2
NM_003412	ZIC1	Zic family member 1

Supplementary Table 2: Primer sequences used in combination with hydrolysis reporter probes (Roche Diagnostics, Germany) for gene expression analysis from amplified RNA (aRNA) samples. These assays target the 3'-region of their respective transcript. Roche Universal ProbeLibrary Assay Design Center available at: https://lifescience.roche.com/shop/en/de/overviews/brand/universal-probe-library

Accession number	Gene symbol	Forward primer	Reverse primer	Probe no.
NM_002275.3	*KRT15*	cattggcatcagggaagc	ttgatgtggaaattgctgct	10
NM_005195.3	*CEBPD*	ggacataggagcgcaaagaa	gcttctctcgcagtttagtgg	64
NM_057088.2	*KRT3*	gaaggcttgtggcctcttg	aattctcgtgactgggcttg	12
NM_015869.4	*PPARG*	aaccaccctgagtcctcaca	tgttccgtgacaatctgtctg	50
NM_000280.3	*PAX6*	agagtgtcgcttccttctaaagtagt	agccaccatacaatatctacttttctc	10
NM_002592.2	*PCNA*	tggcgctagtatttgaagca	cagaaggcatctttactacacagc	2
NM_014491.3	*FOXP2*	agcaaattttaaactgtagcacaaac	cgtcagagaattcaaagtatgaaca	31
NM_014009.3	*FOXP3*	ctgagccctgatccatgc	gccttggatcccaaataaatg	6
NM_005523.5	*HOXA11*	ttggaaagagttagggaaatgc	ggctttcccagatgagatcc	34
NM_004392.5	*DACH1*	tgtgtcaagctttttgcatca	tccacataacaaacaggtgactcta	68
NM_003140.1	*SRY*	ccagctaggccacttaccg	agctttgtccagtggctgtag	71
NM_005986.2	*SOX1*	gagattcatctcaggattgagattcta	ggcctactgtaatcttttctccac	3
NM_003106.3	*SOX2*	gggggaatggaccttgtatag	gcaaagctcctaccgtacca	65
NM_005634.2	*SOX3*	gagaaacatagtatcgggtgagg	aaaacagacgcgacacgac	78
NM_003107.2	*SOX4*	ttaacctgccaccagtgtcc	tcttaaaagcttacagtgtttggcta	52
NM_152989.2	*SOX5*	aaggtatttttcacccctccat	catatggcccacacttctga	39
NM_001145811.1	*SOX6*	tcactgacatgctggactgac	cccaacagccctacacaaac	71
NM_031439.2	*SOX7*	ttcctcaccagccaggtc	atttgcgggaagttgctcta	30
NM_014587.3	*SOX8*	gaggacgcaccctcactc	caaaagttagcgttgcttggt	8

Supplementary Table 2 (contd.)

NM_000346.3	*SOX9*	ttggtttgtgttcgtgttttg	ttcacggagagaacaaaaggtt	23
NM_006941.3	*SOX10*	ccaataacctcattctttgtctga	cgtctcaaggtcatggaggt	25
NM_003108.3	*SOX11*	ggtaaagctacctgaggcagtg	cgaaactttgccatgcattt	88
NM_006943.2	*SOX12*	gggacggacactgacagac	cctgggcataggaagtcaaa	40
NM_005686.2	*SOX13*	tggaagaggttgagaagactcc	ggctgagatccagtccctagt	41
NM_004189.3	*SOX14*	cacgctacggccatgtaa	agaggacagggtgcagacc	23
NM_006942.1	*SOX15*	caacccttctcctgttgga	ctggctatcatgggaggact	34
NM_022454.3	*SOX17*	gctttgaatgtgtcccaaaac	cacacccaggacaacatttct	69
NM_018419.2	*SOX18*	tgccagggttacatttttga	ctggttgtagaaaatacactgcaag	68
NM_007084.2	*SOX21*	cgcttggatttctgacacag	tgaactcagccataagggaaa	68
NM_178424.1	*SOX30*	ccccgacatcaactccttc	tcttcctcctcatcactgtcg	42

Supplementary Table 3: Primer sequences used in combination with hydrolysis reporter probes (Roche Diagnostics, Germany) for gene expression analysis using RNA samples from cell culture experiments. These assays may target any region along their respective transcript.

Accession number	Gene symbol	Forward primer	Reverse primer	Probe no.
NM_057088.2	*KRT3*	tgagctgaagaacatggagga	tcattctcagcagctgtacgtt	31
NM_000223.3	*KRT12*	gacctggagatgcagatcg	cggaagctttggagctcat	17
NM_000526.4	*KRT14*	ctcctctggatcgcagtcat	cgtgcacatccatgacctt	28
NM_002275.3	*KRT15*	gaagagctccgggacaaga	agcctggcattgtcgatct	77
NM_001792.3	*CDH2*	ggtggaggagaagaagaccag	ggcatcaggctccacagt	66
NM_004827.2	*ABCG2*	gcacacaaaagcctactcagc	aacccaggggtaaggaagg	14
NM_005195.3	*CEBPD*	ggacataggagcgcaaagaa	gcttctctcgcagtttagtgg	64
NM_001114980.1	*P63 (ΔN)*	ggaaaacaatgcccagactc	ctgctggtccatgctgttc	45
NM_005547.2	*IVL*	acccatcaggagcaaatgaa	agctcgacaggcaccttct	16
NM_002592.2	*PCNA*	tggcgctagtatttgaagca	cagaaggcatctttactacacagc	2
NM_053056.2	*CCND1*	agaacacggctcacgcttac	cagacaaagcgtccctcaag	71
NM_000389.4	*CDKN1A*	cgaagtcagttccttgtggag	catgggttctgacggacat	82
NM_000076.2	*CDKN1C*	ctcctttccccttcttctcg	tccatcgtggatgtgctg	55
NM_030761.4	*WNT4*	gcagagccctcatgaacct	cacccgcatgtgtgtcag	4
NM_001904.3	*CTNNB1*	gctttcagttgagctgacca	caagtccaagatcagcagtctc	21
NM_002093.3	*GSK3B*	gacatttcacctcaggagtgc	caagtccaagatcagcagtctc	67

Supplementary Table 4: Primary antibodies used for immunofluorescent staining of cryosections of human corneoscleral specimens.

Antigen	Clone	Raised in	Concentration	Supplier
Sox8	4E4.1	Mouse	1:50	Merck Millipore
Sox8	N/A	Rabbit	1:2000	Dr. Sock, Erlangen
Sox9	3C10	Mouse	1:150	BioRad (formerly Serotec)
Sox9	N/A	Rabbit (1)	1:500	Merck Millipore
Sox9	N/A	Rabbit (2)	1:2000	Dr. Sock, Erlangen
Sox10	BC34	Mouse	1:100	Abcam
Sox10	N/A	Rabbit	1:100	DCS Diagnostics
Sox7	S5-1216	Mouse	1:300	BD Pharmingen
Sox17	P7-969	Mouse	1:500	BD Pharmingen
Sox18	N/A	Rabbit	1:300	Sigma
Melan-A	EP1422Y	Rabbit	1:500	Abcam
p75 NGF-R	ME20.4	Mouse	1:100	Abcam
N-cadherin	6G11	Mouse	1:50	DAKO
Keratin 3/76	AE5	Mouse	1:50	Merck Millipore
Keratin 15	LHK15	Mouse	1:500	Abcam
Keratin 15	EPR1614Y	Rabbit	1:1000	Abcam
Oct-4	N/A	Rabbit	1:100	Cell Signaling
P63α	N/A	Rabbit	1:100	Cell Signaling
Pax6	N/A	Rabbit	1:200	Abcam
Ki-67	SP6	Rabbit	1:1000	Abcam
PCNA	PC10	Mouse	1:1000	Abcam
IgG1	Isotype control	Mouse	1:100	BD Biosciences
IgG2a	Isotype control	Mouse	1:150	BD Biosciences
IgM	Isotype control	Mouse	1:50	BD Biosciences
IgG	Isotype control	Rabbit	1:1000	BD Biosciences

Supplementary Fig. 1: Immunohistochemical localization of SoxE family members in corneoscleral tissue sections. Individual channels of double labeling experiments shown in Fig. 2C.

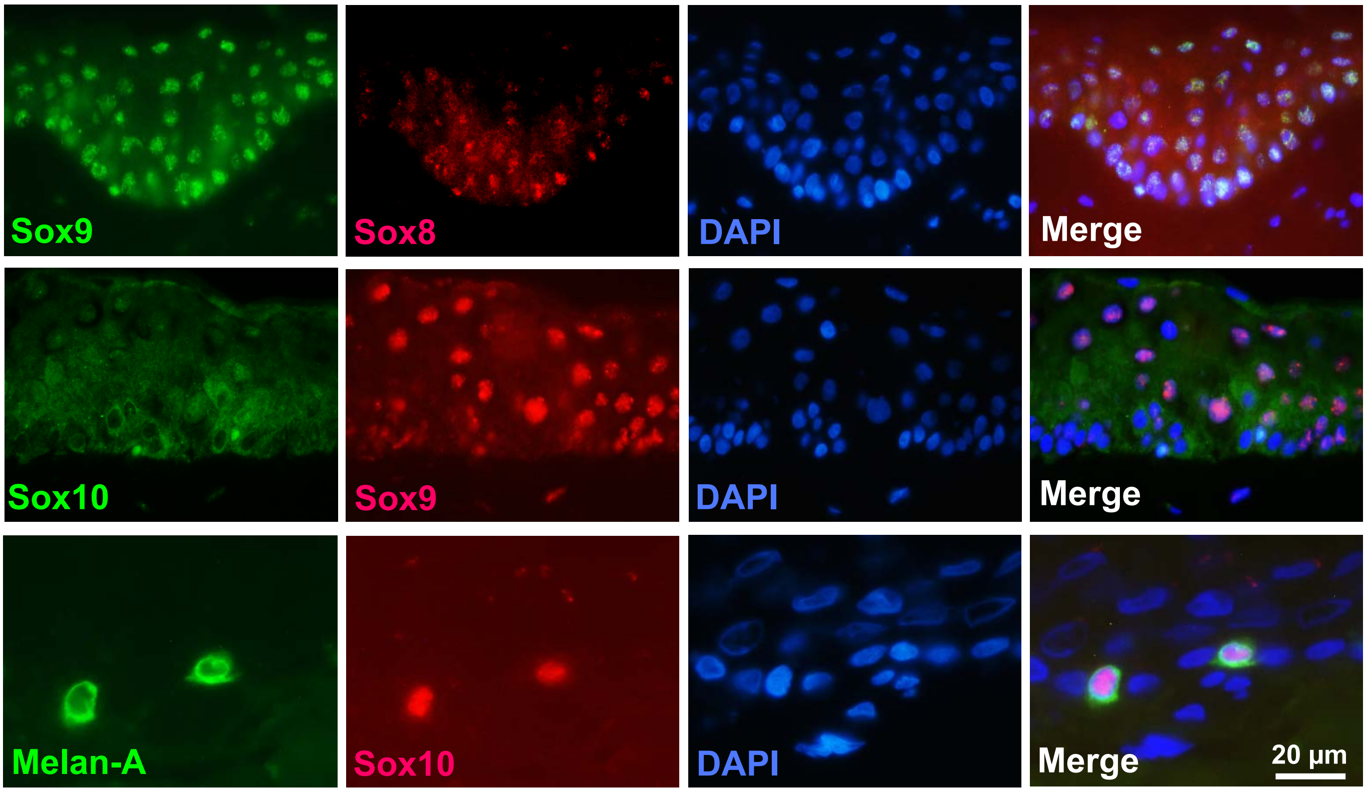

Supplementary Fig. 2: Co-localisation of Sox9 with markers related to progenitor cell phenotype, differentiation and proliferation in the limbal epithelium. Individual channels of double labelling experiments shown in Fig. 3.

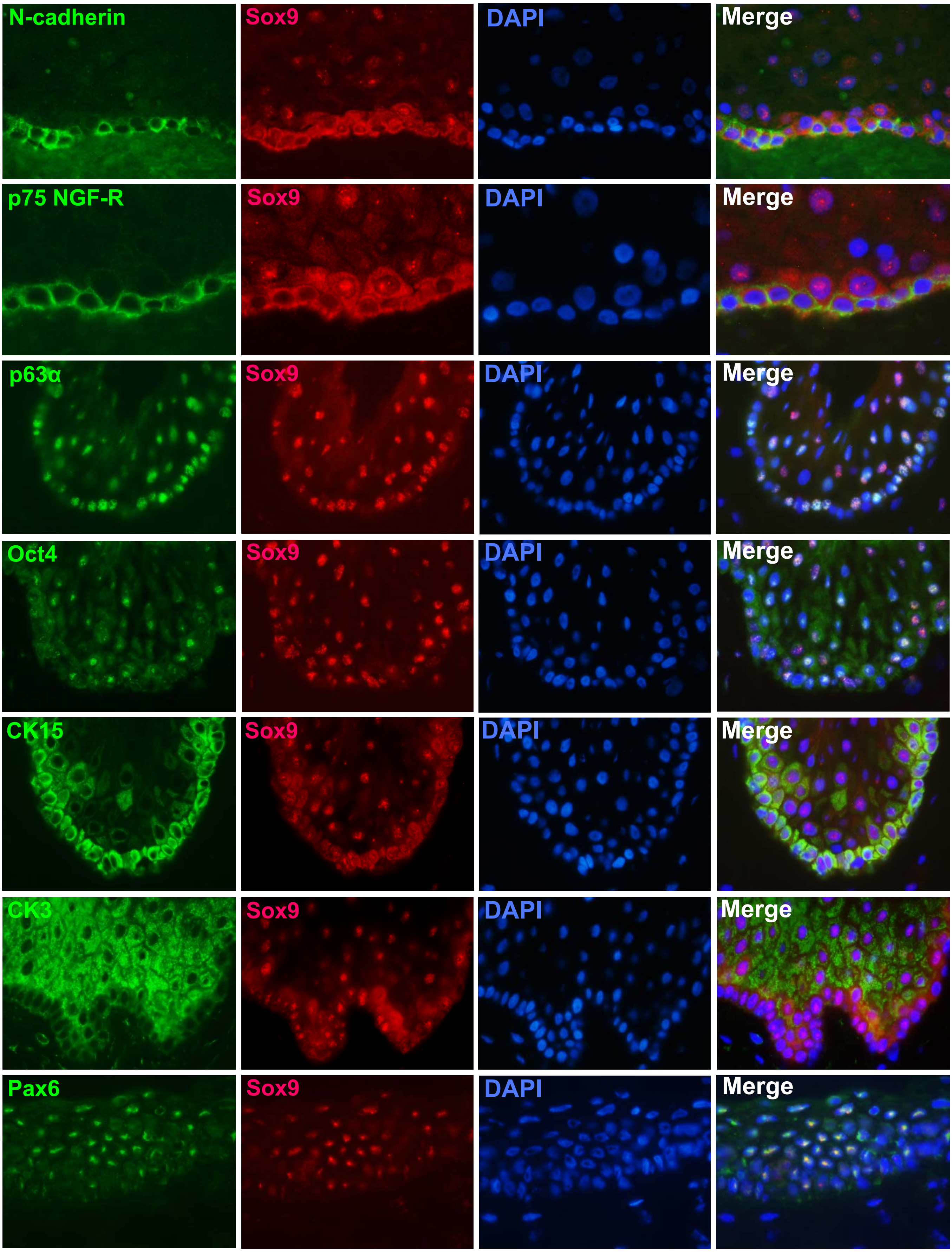

Supplementary Fig. 2: Continued.

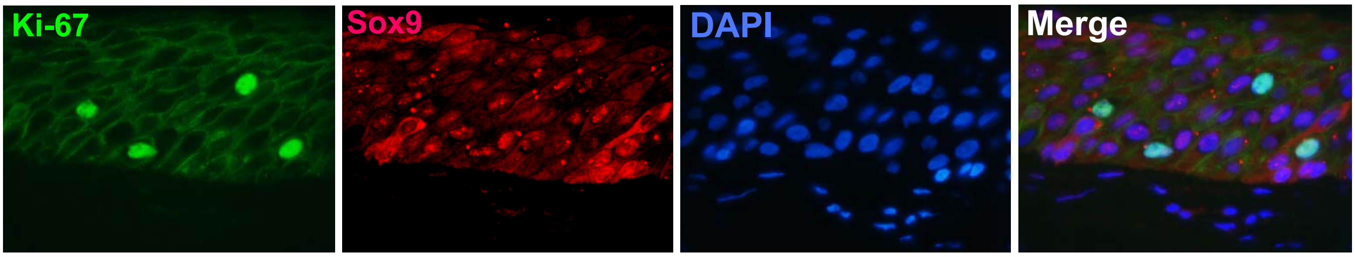

Supplementary Fig. 3: Individual channels of negative control experiments using isotype-specific antibodies.

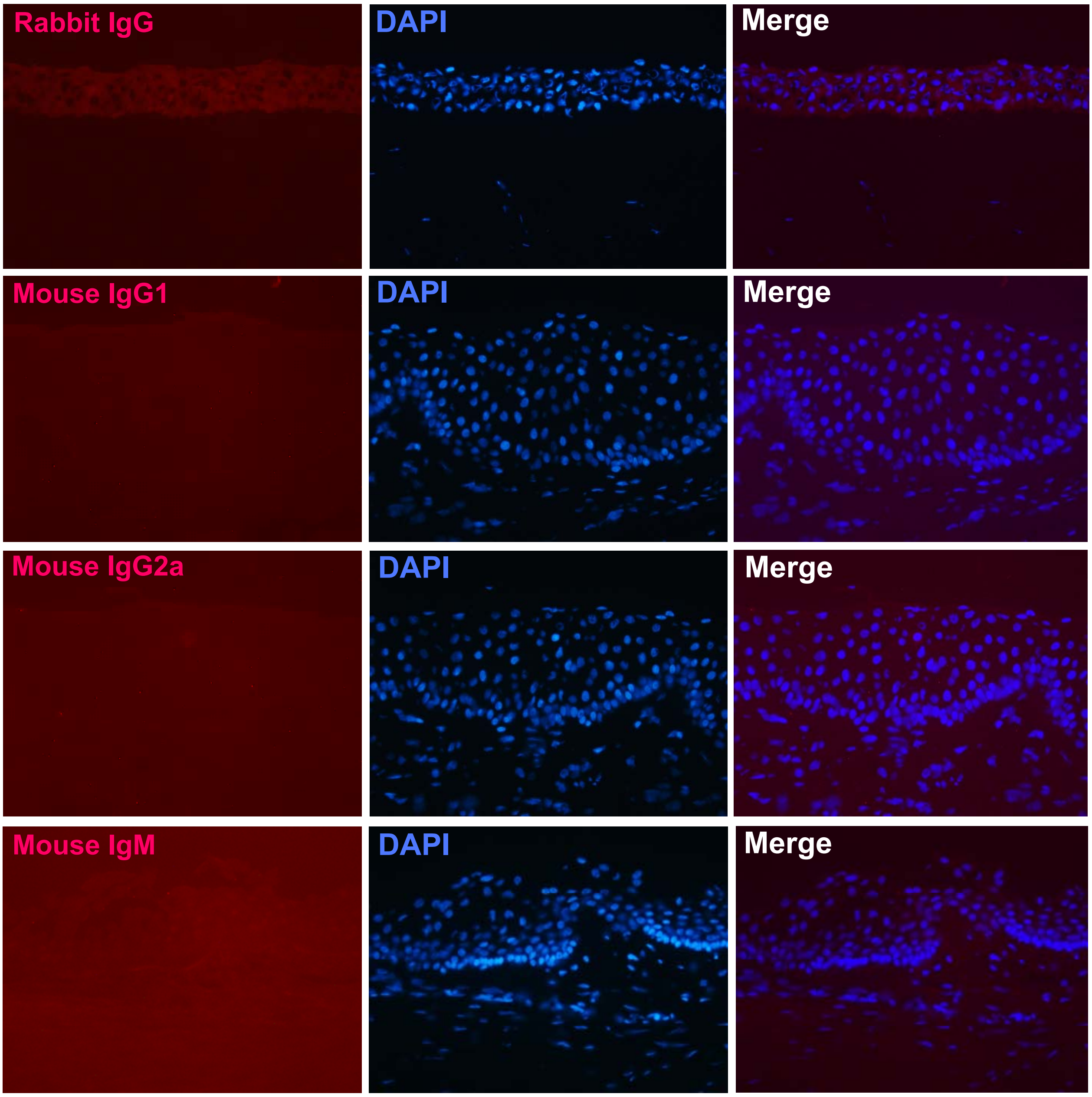

Supplementary Fig. 4: Uncropped versions of all Western blots shown in Fig. 6 and 7.

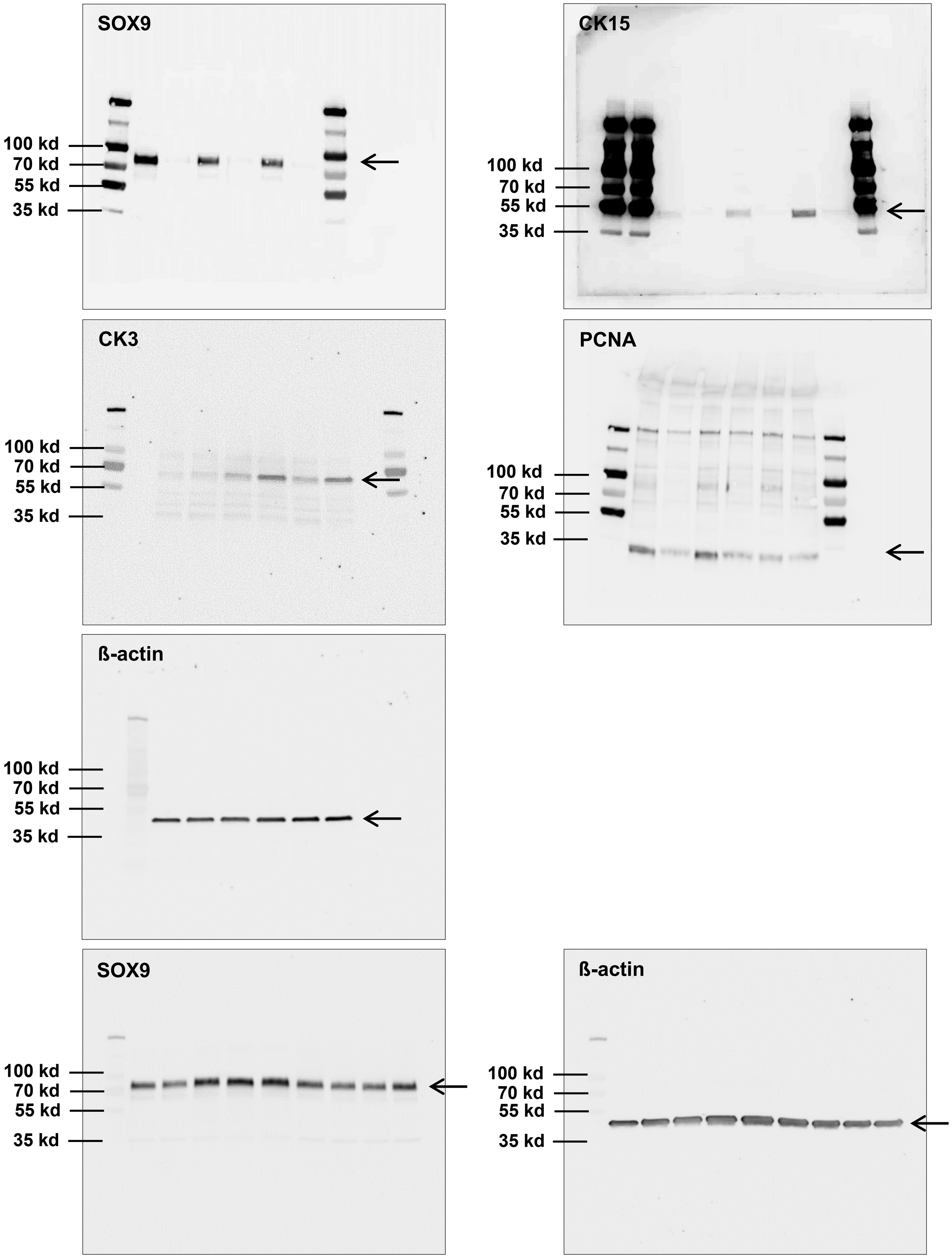

Supplement: Supplementary file 1 — Supplementary information [file 41598_2018_28596_MOESM1_ESM.pdf]
